# Shared mechanisms of enhanced plasmid maintenance and antibiotic tolerance mediated by the VapBC toxin:antitoxin system

**DOI:** 10.1128/mbio.02616-24

**Published:** 2024-12-20

**Authors:** Sarah Hollingshead, Gareth McVicker, Maria R. Nielsen, YuGeng Zhang, Giulia Pilla, Rebekah A. Jones, Jonathan C. Thomas, Sarah E. H. Johansen, Rachel M. Exley, Ditlev E. Brodersen, Christoph M. Tang

**Affiliations:** 1 Sir William Dunn School of Pathology, University of Oxford, Oxford, United Kingdom; 2 Department of Biosciences, Nottingham Trent University, Nottingham, United Kingdom; 3 Department of Molecular Biology and Genetics, Aarhus University, Aarhus, Denmark; University of Wisconsin-Madison, Madison, Wisconsin, USA

**Keywords:** *Shigella*, plasmid stability, antibiotic tolerance, TA systems, VapBC

## Abstract

Toxin:antitoxin (TA) systems are widespread in bacteria and were first identified as plasmid addiction systems that kill bacteria lacking a TA-encoding plasmid following cell division. TA systems have also been implicated in bacterial persistence and antibiotic tolerance, which can be precursors of antibiotic resistance. Here, we identified a clinical isolate of *Shigella sonnei* (CS14) with a remarkably stable pINV virulence plasmid; pINV is usually frequently lost from *S. sonnei*, but plasmid loss was not detected from CS14. We found that the plasmid in CS14 is stabilized by a single nucleotide polymorphism (SNP) in its *vapBC* TA system. VapBC TA systems are the most common Type II TA system in bacteria, and consist of a VapB antitoxin and VapC PIN domain-containing toxin. The plasmid stabilizing SNP leads to a Q12L substitution in the DNA-binding domain of VapB, which reduces VapBC binding to its own promoter, impairing *vapBC* autorepression. However, VapB^L12^C mediates high-level plasmid stabilization because VapB^L12^ is more prone to degradation by Lon than wild-type VapB; this liberates VapC to efficiently kill bacteria that no longer contain a plasmid. Of note, mutations that confer tolerance to antibiotics in *Escherichia coli* also map to the DNA-binding domain of VapBC encoded by the chromosomally integrated F plasmid. We demonstrate that the tolerance mutations also enhance plasmid stabilization by the same mechanism as VapB^L12^. Our findings highlight the links between plasmid maintenance and antibiotic tolerance, both of which can promote the development of antimicrobial resistance.

**IMPORTANCE:**

Our work addresses two processes, the maintenance of plasmids and antibiotic tolerance; both contribute to the development of antimicrobial resistance in bacteria that cause human disease. Here, we found a single nucleotide change in the vapBC toxin:antitoxin system that stabilizes the large virulence plasmid of *Shigella sonnei*. The mutation is in the vapB antitoxin gene and makes the antitoxin more likely to be degraded, releasing the VapC toxin to efficiently kill cells without the plasmid (and thus unable to produce more antitoxin as an antidote). We found that vapBC mutations in *E. coli* that lead to antibiotic tolerance (a precursor to resistance) also operate by the same mechanism (*i.e.*, generating VapB that is prone to cleavage); free VapC during tolerance will arrest bacterial growth and prevent susceptibility to antibiotics. This work shows the mechanistic links between plasmid maintenance and tolerance, and has applications in biotech and in the design and evaluation of vaccines against shigellosis.

## INTRODUCTION

Type II toxin–antitoxin (TA) systems are small two-gene operons abundant on both bacterial plasmids and genomes that have been implicated in plasmid maintenance, virulence, antimicrobial resistance (AMR), and phage defense (1–3). Virulence-associated protein BC (*vapBC*) is the most abundant Type II TA system, accounting for a third of all known loci, and is broadly distributed across bacteria (4). The VapB antitoxin consists of two domains, an N-terminal promoter-binding domain and a C-terminal, intrinsically disordered region, which conceals and inhibits the active site of the VapC toxin in the VapBC complex (5). VapC contains a PilT N-terminal (PIN) domain, with endoribonuclease activity that is capable of cleaving highly specific cellular RNA targets (*e.g.*, tRNA or rRNA) (5-6–8) The intact VapBC complex has a higher-order hetero-octameric structure with a VapB_4_VapC_4_ stoichiometry that exposes two DNA-binding domains that are capable of interacting with the promoter of TA system during transcriptional autoregulation (9, 10).

In *Shigella* spp., a *vapBC* (*mvpAT*) locus is necessary for the maintenance of the ~210 kb pINV virulence plasmid at 37°C (10–13). pINV encodes a Type III Secretion System (T3SS) in a 30 kb pathogenicity island (PAI) that is essential for epithelial cell invasion and virulence of *Shigella* spp. (14–16). VapBC promotes pINV maintenance in *Shigella* by mediating post-segregational killing (PSK) of daughter cells that fail to acquire a plasmid after cell division; without *de novo* VapB production in the absence of pINV, preferential degradation of the VapB leads to free VapC causing cell death/arrested growth. The related *vapBC* locus on the chromosomally integrated *Escherichia coli* F plasmid (17) is mutated in bacteria that are tolerant to beta-lactam antibiotics, before subsequently evolving resistance (18). The VapB antitoxin in enteric pathogens is cleaved by the Lon protease (12, 13, 19), an ATP-dependent, AAA^+^ protease that targets several regulatory proteins, including TA system antitoxins (20) through targeted degradation (21–23). Lon specifically recognizes regions rich in aromatic residues, although a consensus recognition sequence has not been identified (24, 25). Moreover, the activity and specificity of Lon towards particular targets can be affected by other factors including inorganic polyphosphate, a linear polymer of orthophosphate produced in enteric bacteria during stress (26, 27). While studies of Lon-deficient mutants in *Shigella* spp. implicate the protease in degradation of VapB (11), the cleavage site(s) and influence of the structural state of VapB (*i.e.*, isolated VapB, or in VapBC complexes that are free or bound to DNA) on this process remain unknown.

Here, we describe the identification of a clinical isolate of *Shigella sonnei* with a remarkably stable pINV virulence plasmid. We show that high-level pINV stabilization results from a single amino acid substitution (Q12L) in the N-terminal promoter DNA-binding region of VapB. The VapB substitution confers plasmid stabilization at different temperatures (*i.e.*, those found in the human body and environment), and in several bacterial species. Although the VapB Q12L substitution reduces the affinity of VapBC for its promoter, high-level plasmid stabilization does not result from a change in DNA binding or transcriptional regulation. We demonstrate that Lon cleaves VapB at sites adjacent to aromatic residues in the C terminal region of the protein that interacts with and inhibits VapC; VapB is protected from cleavage when in a complex with VapC bound to its promoter. Of note, the Q12L substitution does not introduce new Lon cleavage sites but does promote Lon cleavage of VapB, enhancing the release of VapC and subsequent PSK. We show that the increased cleavage of VapB is responsible for high-level pINV stabilization of the VapB Q12L substitution, which does not stabilise plasmids in *lon*-deficient bacteria. Interestingly, the single nucleotide polymorphism leading to the VapB Q12L substitution maps close to *vapB* mutations in the chromosomally integrated F plasmid that confer tolerance to antibiotics in *E. coli* (18, 28). We demonstrate that VapB amino acid substitutions causing antibiotic tolerance also promote plasmid maintenance through the same mechanism that VapB^L12^ increases PSK, highlighting the mechanistic links between TA-mediated tolerance and plasmid maintenance, which can both promote the emergence of AMR.

## RESULTS

### A point mutation in *S. sonnei* pINV *vapBC* enhances plasmid maintenance

**Fig 1:**
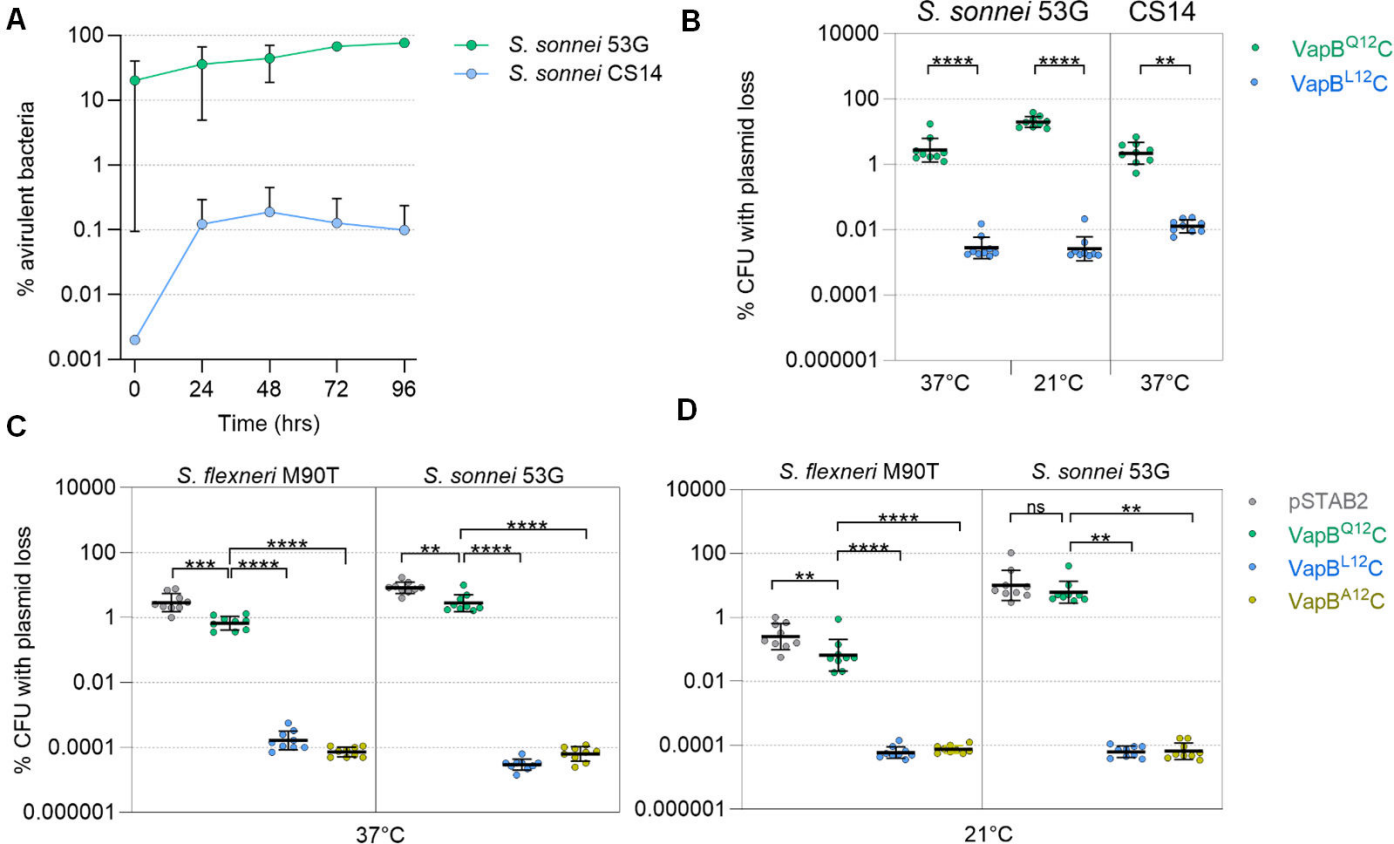
Stabilization of *S. sonnei* CS14 pINV is mediated by a single amino acid substitution in VapB. (**A**) Emergence of avirulent CR^-^ colonies at 21°C during liquid growth of *S. sonnei* 53G and *S. sonnei* CS14 (*n* = 2; error bars, SD). (**B**) The effect of *vapB^Q12^* and *vapB^L12^* in *S. sonnei* 53G and *S. sonnei* CS14 on pINV loss. pSTAB2 loss in *S. flexneri* M90T and *S. sonnei* 53G lacking pINV at 37°C (**C**) or 21°C (**D**). *vapBC* alleles are indicated. Results are from nine colonies from ≥3 independent experiments. Statistical significance determined using Kruskal–Wallis and two-way ANOVA: ****, *P* < 0.0001; ***, P < 0.001; **, *P* < 0.01; ns, *P* > 0.05.

*S. sonnei* frequently becomes avirulent in the laboratory through loss of the pINV plasmid (14, 16, 29). Virulent and avirulent strains of *S. sonnei* can be distinguished by their colony morphology on media containing Congo Red (CR) as virulent bacteria expressing the T3SS bind CR giving rise to red (CR^+^) colonies, while avirulent *S. sonnei* (lacking a T3SS through plasmid loss) do not bind CR and form white (CR^-^) colonies (30). After approximately 25 generations of growth on CR-containing agar at 37°C, around 10% of *S. sonnei* 53G colonies become CR^-^ through loss of pINV (13). We investigated whether *S. sonnei* isolates vary in their rate of plasmid loss by examining the phenotype of 26 clinical isolates on media containing CR. Intriguingly, we noticed that the clinical isolate *S. sonnei* CS14 (31) formed no CR^-^ colonies when plated onto CR-containing agar. In *S. sonnei* 53G, pINV is lost more often during growth at 21°C than 37°C (13). Therefore, we compared the appearance of CR^-^ colonies of wild-type *S. sonnei* CS14 and 53G after growth at 21°C for 96 h. At this point, >99% of CS14 colonies were CR^+^ with ≤0.1% of colonies appearing CR^-^, while >25% of *S. sonnei* 53G colonies were CR^-^ (Fig. 1A). Therefore, we sought to define the mechanisms that led to increased pINV maintenance by *S. sonnei* CS14.

As TA systems can be essential for plasmid maintenance, we speculated that a change in the repertoire of TA systems could stabilize *S. sonnei* CS14 pINV. We determined the whole genome sequence of *S. sonnei* CS14 by short and long read sequencing, and assembled the genome into a single circular 4.8 mb molecule, plus six plasmids (pINV and pCS14-1 to -5), ranging in size from 2092 bp to the 213 kb pINV; pCS14-1 (108.7 kb) carries four antimicrobial resistance genes (*aadA*, *dfrA1*, *SAT*-2, and *sul2*). pINV from *S. sonnei* 53G and CS14 both contain two TA systems, *vapBC* and *relBE.* Inspection of tRNA^fMet^ alleles, the target of VapC, revealed no obvious differences that could account for enhanced pINV maintenance in CS14. However, sequence comparison of the pINV *vapBC* loci in the two strains revealed two single nucleotide polymorphisms (SNPs, Fig. S1) between the strains; a T->C substitution at −24 between the −10 and −35 promoter sites, and an A->T substitution in the *vapB* ORF, resulting in a VapB Q12L substitution in *S. sonnei* CS14. To determine whether the *vapB* SNP is responsible for high-level pINV stability, we swapped the *vapB* alleles on the virulence plasmids in *S. sonnei* CS14 and 53G, introducing VapB^Q12^ into CS14 pINV and VapB^L12^ into 53G pINV, without altering the promoter. For selection, a chloramphenicol resistance cassette (*cat*) was inserted downstream of *vapC*; as controls, we generated strains carrying plasmids with their native *vapB* allele and *cat* at the identical position. The presence of the pINV was monitored at 37°C by placing *sacB-neo^R^* into *mixH* in the T3SS PAI (13). This provides a sensitive method to detect plasmid loss from *S. sonnei* as only bacteria that have lost *sacB* grow on media containing sucrose (13). Remarkably, we found that introduction of VapB^L12^ into pINV reduced plasmid loss in *S. sonnei* 53G at 37°C and 21°C by approximately 1,000- and 10,000-fold, respectively (both *P* < 0.0001; Fig. 1B). Similarly, introduction of VapB^Q12^ into CS14 pINV increased plasmid loss at 37°C by >100 fold (*P* = 0.01; Fig. 1B). We measured the emergence of CR^-^ colonies to assess plasmid loss from *S. sonnei* CS14 at 21°C as sucrose-based selection in this strain was unsuccessful at this temperature. The abundance of CR^-^ colonies of *S. sonnei* CS14 with VapB^Q12^ reached approximately 10% after 25 generations at this temperature, while no CR^-^ colonies were detected when the VapB^L12^ substitution was introduced into the same strain. Together these data demonstrate that VapB^L12^ is sufficient to reduce pINV loss during growth at 37°C and 21°C.

To further dissect the effect of *vapBC* alleles on plasmid maintenance, we employed the model vector pSTAB2 (Fig. S2A) (11), which harbors the origin of replication from pINV and a *sacB-neo^R^* marker for selection/counter-selection (11). We inserted the *vapBC* alleles from *S. sonnei* 53G (expressing VapB^Q12^C) or CS14 (expressing VapB^L12^C) into pSTAB2, in addition to VapB^A12^C, which lacks a side chain at this position. pSTAB2 loss was then monitored in both *S. flexneri* M90T and *S. sonnei* 53G lacking pINV at 37°C and 21°C. Consistent with previous work, we found that the effect of VapBC in stabilizing pSTAB2 varies with temperature (11). In *S. flexneri*, VapB^Q12^C significantly stabilized pSTAB2 at 21°C and 37°C (*P* < 0.001 and *P* = 0.0001, respectively), with 10-fold greater stabilization at 37°C than 21°C (Fig. 1C and D). In *S. sonnei* 53G, also VapB^Q12^C significantly stabilized pSTAB2 at 37°C, but not at 21°C (*P* = 0.0012 and *P* > 0.9999, respectively; Fig. 1C and D). By contrast expression of VapB^L12^C or VapB^A12^C reduced pSTAB2 loss by over 1,000-fold in both *S. sonnei* and *S. flexneri* compared with VapB^Q12^C at 37°C and 21°C (*P* < 0.0001, Fig. 1C and D). In fact, loss of pSTAB2 containing *vapB^L12^C* or *vapB^A12^C* was below the limit of detection, so we were unable to identify differences in plasmid stabilization conferred by these two alleles. Finally, to test the ability of VapB^L12^C to stabilize plasmids in other species, pSTAB2 derivatives expressing VapB^Q12^C or VapB^L12^C were introduced into *E. coli* MG1655. Plasmid loss assays confirmed that VapB^L12^C or VapB^A12^C significantly stabilize pSTAB2 in *E. coli* at 37°C and 21°C compared with VapB^Q12^C (*P* < 0.001; Fig. S2B). Taken together, our data demonstrate that expression of VapB^L12^C dramatically stabilizes plasmids in different species and at different temperatures, with the presence of a small aliphatic amino acid at VapB position 12 (Leu or Ala) increasing plasmid maintenance.

### Reduced affinity of VapB^L12^C for its promoter does not affect plasmid maintenance

**Fig 2:**
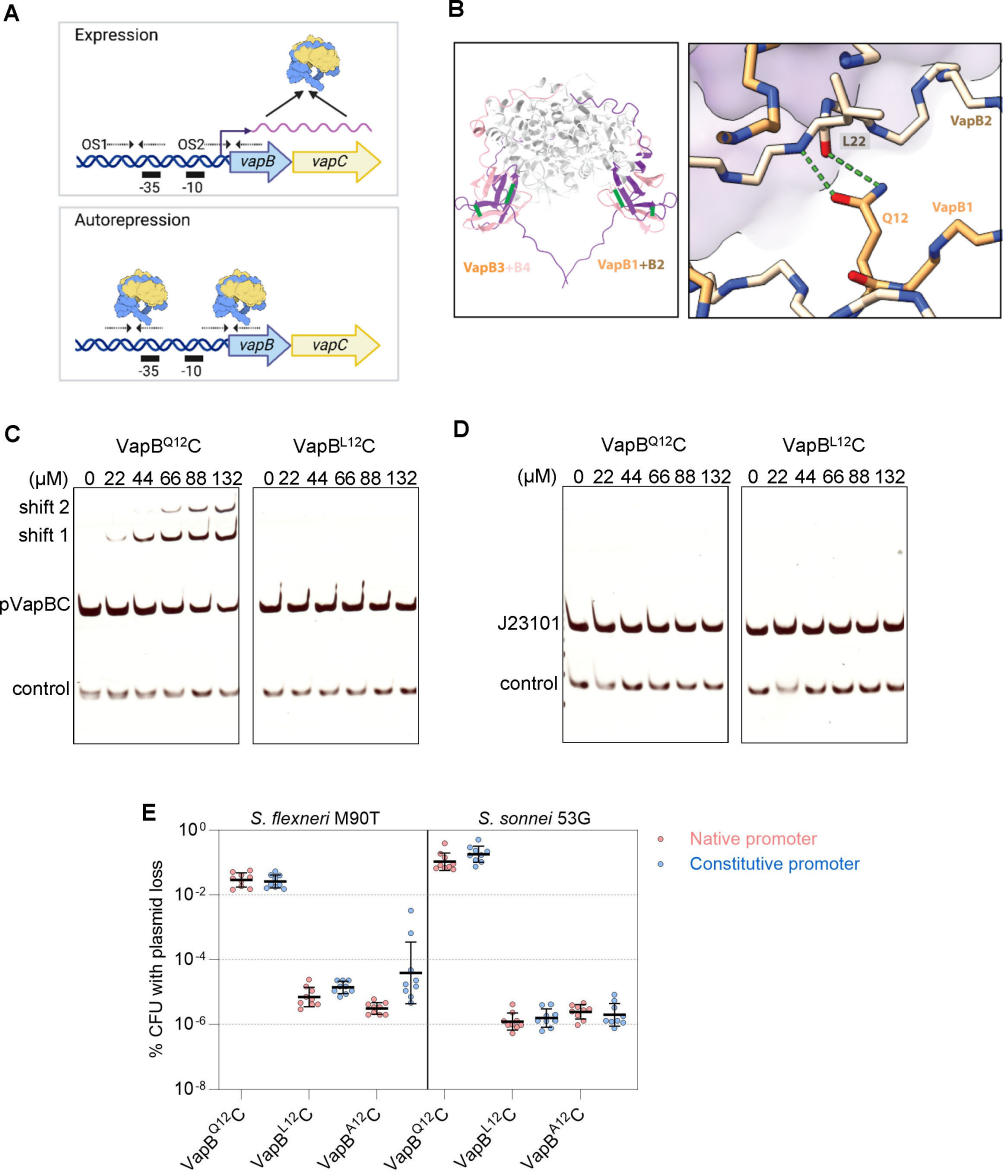
VapB^L12^C has impaired binding to its promoter. (**A**) Schematic of the autorepressed *vapBC* bicistronic operon, including the promoter containing two operator sites, OS1 and OS2 (each a pair of dotted arrows), that are bound by VapBC and overlap the −10 and −35 sequences of the promoter. (**B**) Structure of *S. sonnei* 53G VapBC hetero-octamer (VapC, purple; VapB, gold; PDB ID: 6SD6). The VapB dimers (VapB1 with VapB2, and VapB3 with VapB4) are indicated, with a close-up of the hydrogen bonds between the side chain of residue VapB1^Q12^ and the backbone of VapB2^L22^. (**C**) EMSA demonstrating interactions between VapB^Q12^C or VapB^L12^C and the native *S. sonnei* 53G *vapBC* promoter (227 bp, pVapBC), or (**D**) the 150 bp constitutive promoter, J23101; a 68 bp intergenic sequence was included as a control. (**E**) The effect of autoregulation on pSTAB2 loss in *S. flexneri* M90T or *S. sonnei* 53G lacking pINV. Loss of pSTAB2 expressing different *vapBC* alleles (indicated) under the control of the native *vapBC* promoter (red circles) or the constitutive promoter J23101 (blue circles); *n* = 9 colonies from ≥3 independent experiments. Statistical significance calculated using one-way ANOVA and Kruskal–Wallis. Plasmid loss with the native vs constitutive promoter for all *vapBC* alleles was not significant.

Next, we sought to define the molecular basis of enhanced plasmid maintenance conferred by VapB^L12^C and VapB^A12^C. Like many Type II TA systems, *vapBC* expression is auto-regulated at the transcriptional level through direct binding of the higher-order TA complex to two operator sites (OS1 and OS2) in its promoter (Fig. 2A; Fig. S1) (32). In VapBC hetero-octamers, two distinct SpoVT/AbrB-type DNA-binding domains are formed through dimerization of the N-terminal domains of two molecules of VapB, in a “crossed fingers” configuration (Fig. 2B; Fig. S3) (9). Importantly, in each VapB dimer, the side chain of Q12 in one VapB antitoxin forms a hydrogen bond with the backbone of L22 in the adjacent VapB antitoxin, stabilizing the DNA-binding domain by four sites of interaction in the complete hetero-octamer (Fig. 2B). This suggests that the *vapB* polymorphisms might destabilize the VapB DNA-binding domain and thus affect transcriptional repression by VapBC.

To investigate this hypothesis, we examined whether purified complexes of VapB^L12^C and VapB^A12^C differ from VapB^Q12^C in promoter binding by electrophoretic mobility shift assays (EMSAs) and surface plasmon resonance (SPR). EMSAs were performed with the 227 bp promoter from *vapBC* containing both *vapBC* operator sites (OS1 and OS2, Fig. 2C) or a 33 bp dsDNA with OS1 (Fig. S3B through D). As controls, the constitutive 150 bp J23101 promoter was amplified from the corresponding version of pSTAB2 with primers SH136 and GP68 (Fig. 2D), and a 68 bp intergenic region was generated by annealing primers (SH159 with a reverse complementary primer). EMSAs demonstrate that, as predicted, VapB^Q12^C binds to OS1 and OS2, leading to two shifts, while for both VapB^L12^C and VapB^A12^C complexes, no binding to promoter DNA was observed (Fig. 2C and D; Fig. S3B through D). SPR was performed with 32 bp dsDNA fragments containing only OS1 (primer SH180 and a reverse complementary primer, Fig. S4). We were unable to determine the exact *K*_D_ values by SPR for VapB^Q12^C and VapB^L12^C as the traces did not reach equilibrium (Fig. S4). Nevertheless, SPR data demonstrate that the affinity of VapB^L12^C and VapB^A12^C for OS1 is reduced by approximately 5- to 10-fold, respectively, in agreement with results from EMSAs.

These observations suggest that reduced binding of VapB^L12^C or VapB^A12^C to the promoter could relieve auto-repression, increasing VapBC levels, thereby promoting plasmid maintenance. To test this, we inserted *vapB*^Q12^*C*, *vapB*^L12^*C* or *vapB*^A12^*C* into pSTAB2 under the control of the constitutive promoter J23101 (33), which is not bound by VapBC (Fig. 2C) so would not be subject to auto-regulation. Surprisingly, plasmid loss assays in *S. flexneri* M90T, *S. sonnei* 53G, and *E. coli* showed that the ability of the different versions of VapBC to promote plasmid maintenance was not affected by whether they were under the control of the native or constitutive promoter (*P* > 0.05 for all versions; Fig. 2E; Fig. S2B), demonstrating that the increased plasmid maintenance conferred by VapB^L12^C and VapB^A12^C occurs independent of auto-regulation.

### *vapB* polymorphisms in highly efficient VapBCs promote antitoxin degradation by lon

**Fig 3:**
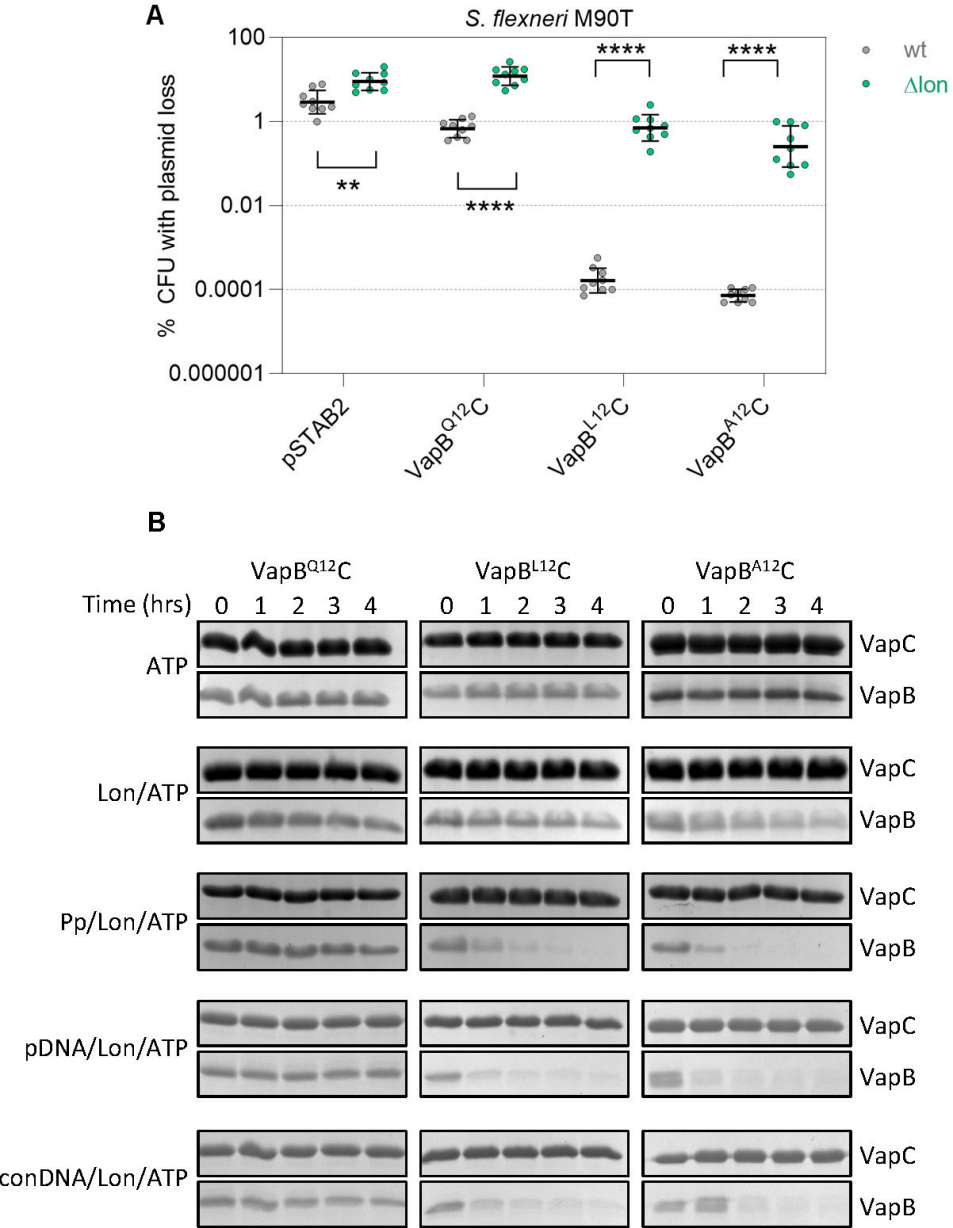
Enhanced degradation of highly efficient VapBCs by Lon protease. (**A**) Stability of pSTAB2 expressing *vapBC* alleles as indicated in *S. flexneri* M90T or *S. flexneri* M90T Δ*lon* (*n* = 9 colonies from ≥3 independent experiments). Statistical significance calculated by two-way ANOVA of *S. flexneri* M90T vs *S. flexneri* M90TΔ*lon* carrying the same vector: **, *P* = 0.0008, ****, *P* < 0.0001. (**B**) *In vitro* proteolysis performed at 37°C with hetero-octameric VapBC as indicated with *S. sonnei* in the presence of ATP, Lon, polyphosphate (Pp), *vapBC* promoter DNA (pDNA), or control DNA (conDNA). Representative result from three independent experiments.

As loss of auto-regulation is not responsible for the effect of the variant *vapB* alleles on plasmid maintenance, we next examined if VapB^L12^ and VapB^A12^ antitoxins are more prone to degradation than VapB^Q12^, which could promote VapC release and activate PSK. As *Shigella* VapB is degraded by Lon (11, 24), we compared the loss of pSTAB2 expressing VapB^Q12^C, VapB^L12^C or VapB^A12^C in *S. flexneri* M90T with or without (*S. flexneri* Δ*lon*) Lon (Fig. 3A) as we were unable to generate a *S. sonnei* Δ*lon* mutant. Results demonstrate that pSTAB2 is lost more frequently in the absence of Lon for all three VapBC variants (*P* < 0.001 for all plasmids comparing *S. flexneri* with or without *lon*; Fig. 3A). Of note, the absence of Lon abolishes VapB^Q12^C-mediated pSTAB2 maintenance, with plasmid loss being almost four orders of magnitude higher for pSTAB2 with *vapB*^L12^*C* or *vapB*^A12^*C* in the *lon* mutant (Fig. 3A), demonstrating that these highly efficient VapBCs depend on Lon for their ability to maintain plasmids

We next assessed whether VapB^L12^C or VapB^A12^C are more sensitive than VapB^Q12^C to cleavage by Lon in *in vitro* degradation assays using purified hexameric *S. sonnei* Lon and VapBC hetero-octamers. Levels of VapB and VapC were followed over time by SDS-PAGE with assays performed in the presence or absence of ATP. Results demonstrate that while VapC is not degraded significantly during the course of assays, VapB is degraded by Lon in an ATP-dependent manner as expected (Fig. 3B; Fig. S5). However, there was no significant difference in the degradation of VapB^Q12^, VapB^L12^ or VapB^A12^C initially (*P* > 0.05; Fig. 3B; Fig. S5), although after 2 h, VapB^A12^ was degraded significantly more than the others (*P* ≤ 0.03; Fig. S5). Polyphosphate can influence Lon activity and alter substrate specificity *in vitro* (26). Therefore, proteolysis assays were repeated in the presence of long-chain polyphosphate (average chain length, 700 units). We found that polyphosphate led to a shift in the pattern of VapB degradation by Lon (Fig. 3B). Addition of polyphosphate significantly reduced Lon degradation of VapB^Q12^ (*P* = 0.0338, after 1 h) while at the same time enhancing Lon degradation of both VapB^L12^ and VapB^A12^ (*P* ≤ 0.0058 and *P* < 0.0001 after 2 hours, respectively; Fig. 3B; Fig. S5).

As VapBC complexes can bind promoter DNA (Fig. 2A; Fig. S3A), we hypothesized that the presence of the *vapBC* promoter could sterically inhibit the ability of Lon to degrade VapB when it is bound to DNA as a hetero-octomer with VapC. To test this, we performed proteolysis assays in the presence of DNA containing the two VapBC operator sites (promoter DNA, pDNA, Fig. 3B; Fig. S5) or control DNA (conDNA, from a non-promoter intergenic region). Of note, we observed virtually no degradation of VapB^Q12^ in the presence of promoter DNA over the course of experiments (Fig. 3B; Fig. S5), while it was degraded in the presence of control DNA (Fig. 3B; Fig. S5). As expected, neither promoter nor control DNA affected Lon degradation of VapB^L12^C or VapB^A12^C which exhibit reduced DNA binding (Fig. 3B; Fig. S5B).

**Fig 4:**
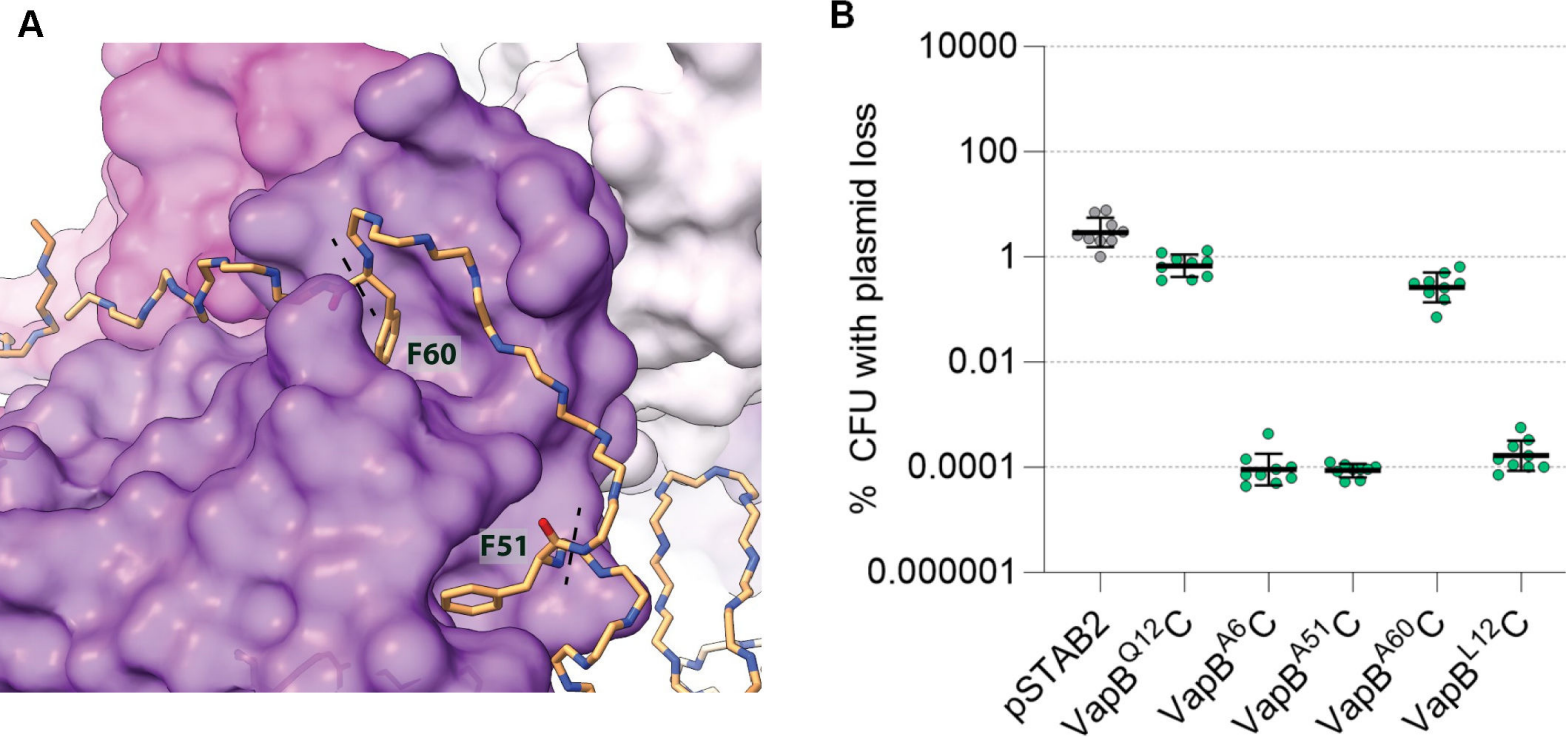
Lon cleavage sites in VapB. (**A**) Lon cleaves VapB next to residues F51 and F60 located in the C-terminal arm of VapB (backbone with Phe side chains) that inhibits VapC (purple, space filling). Lon cleavage sites on the C-terminal side of F51 and F60 are indicated by dashed lines. (**B**) The effect of introducing point mutations F6A, F51A, and F60A into VapB^Q12^C or VapB^L12^C on pSTAB2 loss in *S. flexneri*. Each strain was tested in three independent experiments.

As Lon cleavage sites in antitoxins are largely uncharacterised, we used mass spectrometry to identify sites of Lon cleavage in VapB. Each VapB variant (*i.e.*, VapB^Q12^, VapB^L12^, and VapB^A12^) in complex with VapC was digested by Lon and then by trypsin, and the resulting peptide fragments were analyzed by mass spectrometry. The non-tryptic peptides map Lon cleavage sites on the C-terminal side of Phe6, Phe51, and Phe60 (Fig. S6). Phe51 and Phe60 are towards the C terminus of VapB, where it interacts with VapC (Fig. 4A; Fig. S1), while Phe6 is in the DNA-binding domain. To further confirm the Lon cleavage sites, VapB variants were digested with Lon alone and the mass spectra were manually inspected for the presence of diagnostic peptides that would be generated after Lon cleavage. Charge series for the two peptides indicative of cleavage sites at Phe51 and Phe60 were identified in all VapB variants only in the presence of Lon (Fig. S7 and S8). The putative Lon cleavage site at Phe6 could not be confirmed by manual inspection of mass spectra, due to the small size of the diagnostic peptide. Therefore, mass spectrometry data does not provide evidence that VapB^L12^ and VapB^A12^ have different Lon cleavage sites compared with VapB^Q12^. To assess the relevance of Lon cleavage sites, we generated a set of pSTAB2 vectors expressing VapBC in which Phe60, Phe51, or Phe6 was substituted with alanine. The resulting plasmid loss assays were unexpected as VapB^A6^C and VapB^A51^C significantly stabilized pSTAB2 in *S. flexneri* (*P* ≤ 0.0003 in comparison to pSTAB2 expressing VapB^Q12C^), while the introduction of F60A did not alter plasmid stability (Fig. 4B). In the structure of *S. flexneri* VapBC, Phe6 and Phe51 form hydrophobic interactions that could stabilize the VapB DNA-binding domain (for VapB^F6^), and VapB:VapC interactions (for VapB^F51^). Thus, alanine substitution of these phenylalanine residues likely destabilizes the VapBC hetero-octamer, potentially releasing VapC and promoting plasmid maintenance. In summary, *in vitro* proteolysis assays demonstrate that Lon cleaves to the C-terminus of Phe residues in VapB, but when in a complex with VapC bound to its promoter, VapB is protected from cleavage. VapB^L12^ and VapB^A12^ are significantly more prone to Lon degradation than VapB^Q12^,and this is enhanced in the presence of polyphosphate. However, we did not identify any new cleavage sites in the plasmid-stabilizing substitutions of VapB.

### *vapBC* mutations conferring antibiotic tolerance also enhance plasmid maintenance

**Fig 5:**
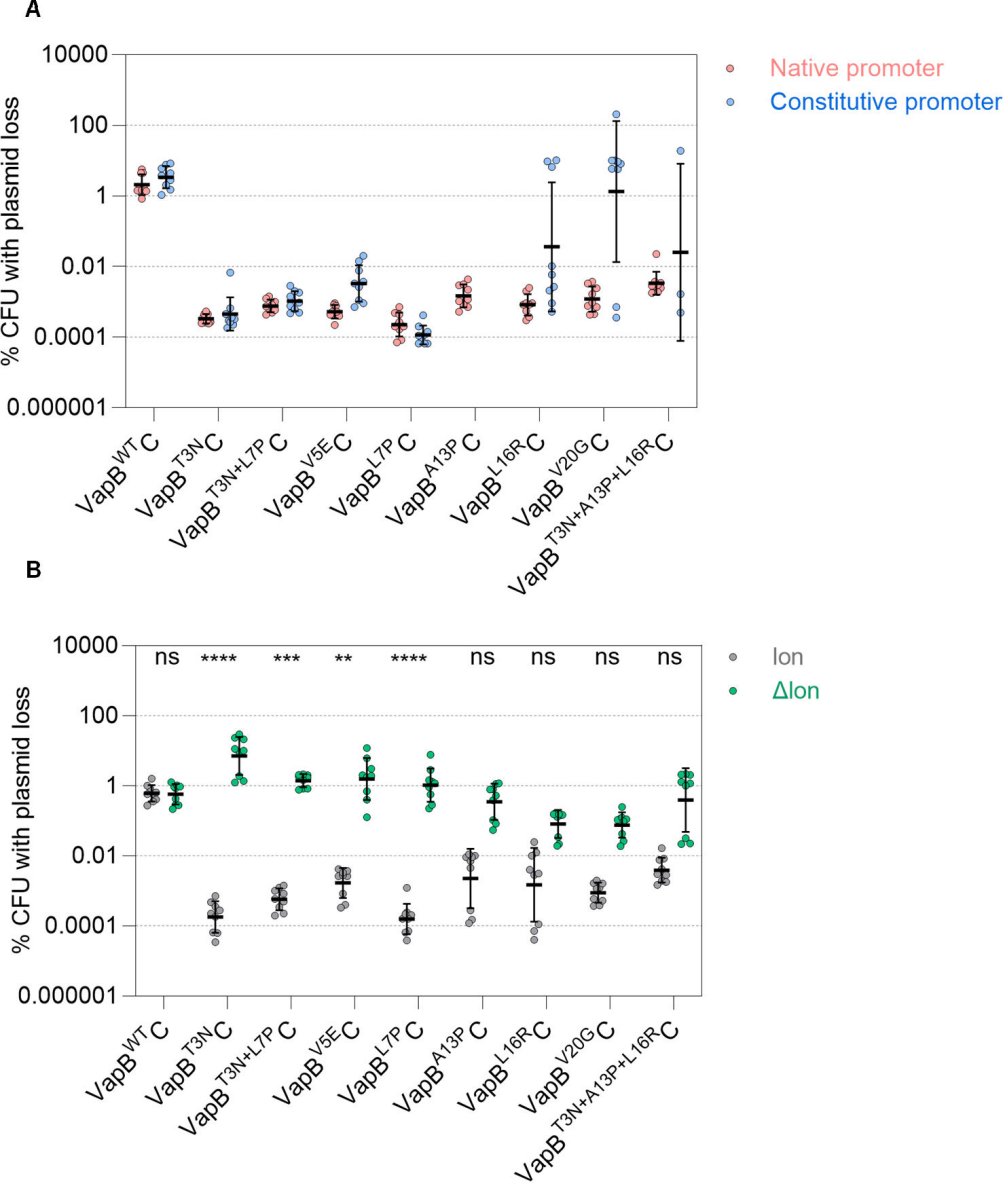
Antibiotic tolerance mutations in *E. coli vapB* affect post-segregational killing. (**A**) Loss from *S. sonnei* of pSTAB2 with different *vapBC* alleles expressed under the native *vapBC* promoter or a constitutive promoter (J23101). Each strain was tested in three independent experiments (except VapB^T3N+A13P+L16R^C with a constitutive promoter, which was tested once). Plasmid loss is reduced with all tolerance mutations compared with VapB^Q12^C (VapB^WT^C) with the native vs constitutive promoter was not significant (ns, *P* > 0.05) for all *vapBC* alleles. (**B**) pSTAB2 loss from *S. flexneri* M90T with (gray circles) or without *lon* (green circles). VapB^T3N^C, VapB^T3N+L7P^C, VapB^V5E^C, and VapB^L7P^C reduce plasmid loss in a Lon-dependent manner, while the absence of Lon does not have a significant impact for VapB^A13P^C, VapB^L16R^C, VapB^V20G^C, and VapB^T3N+A13P+L16R^C. Each strain was tested in three independent experiments, and statistical significance was calculated using Kruskal–Wallis: ****, *P* < 0.0001; ***, *P* = 0.0004; **, *P* = 0.0092.

Mutations that confer *E. coli* tolerance to antibiotics map to the N terminus of VapB (VapB^T3N^, VapB^T3N+L7P^, VapB^V5E^, VapB^L7P^, VapB^A13P^, VapB^L16R^, VapB^V20G^, and VapB^T3N+A13P+L16R^) in *vapBC* on the chromosomally integrated F plasmid (18, 28). To investigate if the substitutions also affect plasmid maintenance, each of the corresponding mutations was introduced into *S. sonnei vapBC* with its own promoter in pSTAB2 and transformed into *S. sonnei* 53G lacking pINV. Interestingly, plasmid loss assays show that all substitutions that cause antibiotic tolerance also reduce plasmid loss compared with VapB^Q12^C (all *P* < 0.0001, Fig. 5A). Similar to VapB^L12^ and VapB^A12^ (Fig. 2C), the tolerance mutations also reduce VapBC binding to its promoter based on a reporter assay (Fig. S9A), which prompted us to test whether the increased level of plasmid maintenance is dependent on its promoter. Therefore, the native *vapBC* promoter in pSTAB2 harboring the *vapBC* tolerance mutations was replaced with the J23101 constitutive promoter, and plasmid loss assessed. All *vapB* tolerance mutations still conferred low level of plasmid loss under the control of the constitutive promoter (Fig. 5A), indicating that *vapB* tolerance mutations do not prevent plasmid loss through loss of auto-regulation, similar to VapB^L12^C and VapB^A12^C. Of note, plasmids expressing VapB^L16R^, VapB^V20G^, and VapB^T3N+A13P+L16R^ under the constitutive promoter showed marked heterogeneity in the level of plasmid loss, and therefore the statistical significance of the results was not analyzed. We found that colonies exhibiting high level plasmid loss had acquired *vapC* loss-of-function mutations (explaining plasmid loss), while *vapC* was unaltered in colonies with low/undetectable plasmid loss (Table S5). No colonies with VapB^A13P^C expressed from a constitutive promoter could be isolated despite multiple attempts, presumably because of high toxicity.

Finally, we tested whether the increased level of plasmid stability induced by *vapB* tolerance mutations was Lon-dependent. pSTAB2 with different *vapBC* variants was introduced into wild-type and Δ*lon S. flexneri*. Plasmid loss assays demonstrate that reduced plasmid loss conferred by VapB^T3N^, VapB^T3N+L7P^, VapB^V5E^, VapB^L7P^, and VapB^Q12L^ is Lon-dependent, similar to VapB^L12^ and VapB^A12^ (Fig. 5B). Interestingly, tolerance mutations that map towards the C terminal end of DNA binding region of VapB (i.e., *vapB*^A13P^, *vapB*^L16R^, *vapB*^V20G^ and *vapB*^T3N+A13P+L16R^) conferred plasmid stability with only partial Lon dependence (Fig. 5B); these tolerance mutations also affect plasmid stability in a Clp-independent manner (Fig. S9B), indicating that their effect does not rely on the activity of other cellular proteases. In summary, all tolerance mutations tested increase plasmid maintenance. Most mutations conferred plasmid stabilization in a Lon-dependent manner, similar to *vapB*^L12^ in the highly stable pINV from *S. sonnei* CS14, with a few mutants exhibiting partial Lon dependence.

### VapB tolerance mutations do not affect the integrity of the VapBC TA complex

**Fig 6:**
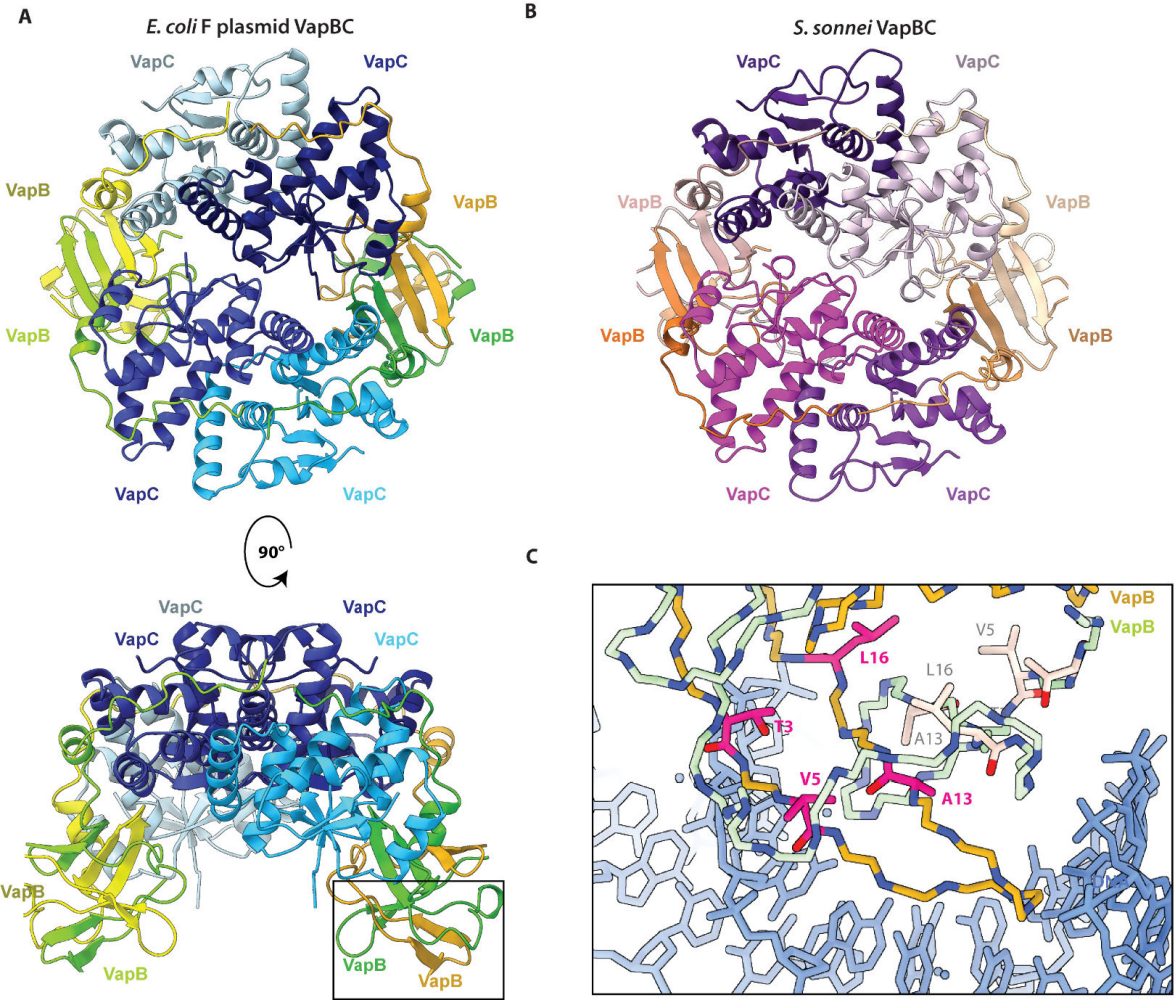
Impact of *vapB* tolerance mutations on the VapBC structure. (**A**) The structure of hetero-octameric VapBC encoded on the *E. coli* F plasmid (top and side view) and (**B**) *S. sonnei* pINV (top view; PDB: 6SD6) (10). The hetero-octameric structure and overall folds are conserved between this and the structure of F plasmid VapBC. (Cα rmsd 4.2–4.7 Å over 132 atoms for each VapC; Cα rmsd of 103–107 Å over 68 atoms for each VapB; Cα rmsd 6.2 Å over 264 atoms for the half complex.) (**C**) Close-up view of the VapB DNA-binding domain (box in panel A). The DNA-binding domain of VapB has been aligned on top of DNA (blue) from the VapBC:DNA structure (PDB: 6IFM) (34). Structures solved with residues altered by tolerance mutations are shown in magenta.

To investigate the structural consequences of the *vapB* tolerance mutations, we used X-ray crystallography to determine structures of several VapB variants in complex with VapC. Initially, the structure of wild-type *E. coli* F plasmid VapBC (VapB^WT^C) was determined to 2.8 Å, which revealed a hetero-octameric complex structurally highly similar to previous structures of *S. flexneri* VapBC (PDB: 3TND) and *S. sonnei* (PDB: 6SD6) (Fig. 6A and B) (9, 10). Moreover, the structures of VapB^T3N^C (2.6 Å), VapB^T3N+A13P+L16R^C (2.8 Å), and VapB^V5E^C (3.2 Å) revealed complexes that are virtually identical to the wild-type complex (Fig. S10). This demonstrates that the overall architecture and integrity of the VapBC hetero-octamer are unaffected by the tolerance mutations. However, we noticed that the DNA-binding domain of VapB appears to be quite flexible and with low local resolution, which means that side chains are not visible in the regions containing the mutations (Fig. S10). Comparison to the structure of DNA-bound *S.* Typhimurium VapBC (PDB: 6IFM, Fig. 6C) (34) shows that the residues altered in all *vapB* tolerance mutants are in the DNA-binding domain of VapB. Of these, Thr3 is close to DNA backbone and could interact directly by hydrogen bonding; substitution to Asn could therefore potentially affect this interaction due to its longer aliphatic side chain. The hydrophobic residues Val5 and Leu16 both point towards the internal, hydrophobic core of the DNA-binding domain (Fig. 6C). As both residues are changed to polar/charged residues upon mutation (*i.e.*, V5E and L16R), this could destabilize the fold of the domain affecting DNA interaction. Ala13 is located close to a loop, and due to low, local resolution this loop cannot be placed with high confidence in the electron density map. A proline at this position, however, could make the protein backbone more rigid and potentially disrupt the fold of the loop, which is likely important for DNA binding (Fig. 6C). In conclusion, the potential tolerance mutations of VapB do not appear to affect the ability of VapBC to form the canonical hetero-octameric structure.

## DISCUSSION

Here, we characterized a clinical isolate of *S. sonnei* with a highly stable virulence plasmid, and identified a plasmid stabilising substitution in the VapB antitoxin of its VapBC TA system. The substitution (Q12L) is in the DNA-binding domain of VapB, and leads to reduced binding of the VapBC complex to its promoter, reducing auto-repression of this TA system. However, this is not responsible for the effect of this substitution on plasmid stabilization. Instead, VapB^L12^ is prone to cleavage by the cellular protease Lon, with its ability to confer plasmid stability dependent on this protease. Furthermore, mutations that confer tolerance to beta-lactam antibiotics also map to the VapB DNA-binding domain, close to VapB^L12^. We found that tolerance mutations also confer plasmid stabilization through the same pathway as VapB^L12^, highlighting the mechanistic links between plasmid maintenance *via* PSK and tolerance to antibiotics.

We discovered two sites of Lon cleavage in VapB to the C-terminal side of Phe51 and Phe61. This is consistent with data indicating that Lon preferentially cleaves sequences rich in aromatic residues (24, 25). We found no evidence that the highly efficient versions of VapBC present novel Lon cleavage sites. However, we cannot exclude this possibility, as Lon could cleave adjacent to Phe7 in the VapB DNA-binding domain, but the seven amino-acid product could be missed by MS; the highly efficient versions of VapBC might be more prone to cleavage at this site as their VapB DNA-binding domains are more disordered (Fig. 6), potentially rendering them prone to proteolysis.

We found that Lon cleaves VapB despite the presence of VapC, which is similar to other TA systems, such as *hipBA* (35), indicating that Lon can recognize and cleave the antitoxin in hetero-octomeric complexes of VapBC. In contrast, in the *ccdAB* TA system, sequestration of the CcdA antitoxin by the toxin CcdB prevents Lon degradation of CcdA (25). Nevertheless, we show that VapB, similar to HipA, can be sequestrated away from Lon degradation through DNA binding (Fig. 3B) (25, 35). Despite this, the increased plasmid stabilization conferred by VapB^Q12L^C was not dependent on its reduced binding to the *vapBC* operator sequence and was still evident when expression of the TA system was driven by a heterologous promoter.

In addition to promoter DNA, we found that polyphosphate modulated Lon cleavage of VapB. The addition of polyphosphate altered the substrate specificity of Lon *in vitro*, further enhancing Lon cleavage of VapB^L12^ and VapB^A12^ compared with VapB^Q12^. We could not identify structural homology between Lon (PDB ID: 6U5Z) and other polyphosphate-binding domains, the C-terminus of PPX1 (36) or the CHAD domain (37) (PDB ID: 2FLO and 3E0S, respectively), suggesting that Lon may bind polyphosphate *via* an as yet uncharacterized mechanism. However, evidence indicates that Lon interacts with polyphosphate through the same domain as it interacts with DNA (38). Importantly, our data highlights that polyphosphate modulates both the substrate specificity and proteolytic activity of Lon on similar variants of the same protein, which has implications for how SNPs influence the Lon activity when polyphosphate is present in bacteria, for example during nutritional stress.

We found that *vapBC* mutations in the integrated F plasmid in *E. coli* that confer antibiotic tolerance also enhance plasmid maintenance. All tolerance mutations map to the N-terminal, DNA-binding region of VapB. We found that the tolerance mutations do not affect the overall structure of the VapBC hetero-octameric complex and enhance plasmid maintenance in a Lon-dependent manner similar to VapB^L12^. Of note, some substitutions (VapB^A13P^, VapB^L16R^, VapB^V20G^, and VapB^T3N+A13P+L16R^) enhanced plasmid maintenance even in the absence of Lon. These substitutions are located towards the body of the complex where VapB interacts with VapC, so they might exert their effect by reducing interactions between VapB and VapC; this is supported by the recovery of *vapC* loss of function mutants when attempting to clone *vapB* variants encoding VapB^L16R^, VapB^V20G^, and VapB^T3N+A13P+L16^ into pSTAB2.

All the *vapB* mutations uncovered through their effect on plasmid maintenance or antibiotic tolerance reduce VapBC binding to its promoter. This makes VapB more prone to degradation, as Lon cleavage of the antitoxin is impaired when the VapBC complex is bound to its promoter (Fig. 3B). The release of VapC from DNA-bound VapBC will not be relevant to death due to PSK, which affects cells that lack a plasmid following cell division; this is supported by our data showing that *vapB* mutations still affect plasmid loss when *vapBC* is expressed by a constitutive promoter (Fig. 2E). However, further studies are needed to establish whether lowered VapBC binding to its promoter on the integrated F plasmid is sufficient to lead to antibiotic tolerance.

Based on clinical severity, antibiotic resistance and disease burden, there is an urgent need for the development of vaccines against shigellosis (39, 40). *S. sonnei* accounts for ~23% of shigellosis worldwide (39) and is particularly prone to develop antibiotic resistance by acquisition of AMR plasmids (41–45). Unfortunately, vaccine development is limited by the high proportion of *S. sonnei* cells that spontaneously lose pINV during growth. For example, vaccines containing live-attenuated strains and lipopolysaccharide O-antigens both require expression of plasmid-encoded antigens by *S. sonnei* (46). We have constructed an attenuated *Shigella* vaccine strain with the pINV stabilizing VapB^L12^ substitution and found that this does not impair bacterial interaction with epithelial cells or their ability to invade cells in enteroids (47). This highlights the potential translational benefits of understanding *S. sonnei* plasmid dynamics, which could also allow development of clinically relevant animal models of shigellosis.

In summary, we identify single substitutions in the antitoxin of the common Type II TA system, VapBC, that are important for maintenance of the pINV plasmid in *Shigella* spp. and the development of tolerance against antibiotics. The substitutions have their effects on these distinct processes through a common mechanism, the enhancement of cleavage of the antitoxin by the house-keeping protease, Lon, and highlight that dissociation of multimeric VapBC from DNA is not necessary for plasmid maintenance. Our findings provide novel insights into the evolution of antimicrobial resistance, which usually requires bacteria becoming tolerant to the drug, and could be exploited for vaccine and biotechnical applications by allowing plasmid maintenance without the additional use of antibiotics.

## MATERIALS AND METHODS

### Strains and growth media

Bacterial strains and plasmids used in this study are shown in Tables S1 and S2, respectively. *E. coli* and *Shigella* spp. were propagated in Luria broth (LB, Invitrogen) or on solid media containing 1.5% (w/v) agar (Oxoid). Antibiotics were used at the following concentrations: carbenicillin, 50 µg mL^−1^; chloramphenicol, 34 µg mL^−1^; kanamycin, 50 µg mL^−1^. Screening for CR^+^ and CR^-^ colonies was performed on tryptic soy broth (TSB, Fluka) solid media containing 0.01% (w/v) CR (Sigma). Sucrose selection was performed on solid media containing 1% (w/v) tryptone (Fluka), 0.5% (w/v) yeast extract (Fluka), 1.5% (w/v) agar, and 10% (w/v) sucrose.

### Strain and plasmid construction

DNA manipulations were performed in *E. coli* DH5α. Plasmids were assembled by restriction digestion and ligation, or with NEBuilder HiFi DNA assembly master mix (New England Biolabs). Primers used in this study are shown in Table S3. Electroporation was used to transform *Shigella* with plasmids or linear DNA, while chemically competent *E. coli* was used for transformation with plasmid DNA. To introduce mutations into *Shigella* spp., λ Red recombination (48) was performed using PCR products including ~ 1 kb of upstream and downstream sequence. Following λ Red recombination in *S. flexneri*, P1*vir* transduction was used to transfer mutations into a new genetic background (13); the *cat* cassette was excised using the FLP recombinase encoded by pCP20 (48). For inserting mutations into the *S. sonnei* chromosome, pCONJ4s was employed (49), using 500 bp of upstream and downstream flanking sequence, with *E. coli* λ*pir* as the donor. Fragments were amplified from *S. sonnei* 53G genomic DNA and inserted into pET28a using *Nde*I/*Xho*I digestion. Constructs expressing modified VapBs were generated by overlap PCR and confirmed by sequencing.

### Sequencing library preparation and assembly

Nanopore sequencing libraries were prepared using the ligation sequencing kit SQK-LSK108 (Oxford Nanopore Technologies) and native barcodes (EXP-NBD103). The library was loaded on an R9.4.1 MinION flowcell. Nanopore reads were base called using the high-accuracy model of guppy v3.6.1 and end and middle adapters were trimmed using Porechop v0.2.4 with 95% and 85% matches to adapters, respectively. Nextera XT Illumina sequencing libraries were prepared according to manufacturer’s instructions. Libraries were sequenced using 2 × 250 bp paired-end sequencing reads with v2 chemistry on an Illumina MiSeq. Adapters were trimmed from raw reads using trimmomatic v0.39 (50).

A hybrid assembly was produced from Nanopore/Illumina data by first assembling overlapping trimmed Nanopore long-reads into a scaffold using Canu v2.2 (51). The assembled genome sequence was first polished with nanopore data *via* four iterations of Racon v.1.5.0 (52) (with parameters -m 2 -g -2 -u), followed by Medaka v1.8.0 (53) and Nanopolish v0.13.2 (54). Resulting contigs were subsequently polished with Illumina data using both Racon and Pilon v.1.2.3 (55). All contigs were manually trimmed, while the chromosomal molecule was reoriented, so the molecule began with *dnaA*. Plasmids were screened for the presence of antimicrobial resistance genes using CARD v3.2.9 (56) and average mean read depths for each molecule.

### Protein purification

VapBC and VapB were purified as previously (9). Following Ni affinity chromatography, proteins were passed over an S200 10/30 Increase column equilibrated with 50 mM Tris pH 8.0, 300 mM NaCl and 1 mM dithiothreitol (DTT). His-MBP tagged Lon was expressed in *E. coli* B834. Cultures were grown at 37°C to an OD_600_ ~ 1, induced with 1 mM IPTG and incubated at 21°C for 3 h prior to harvesting. Cell pellets were resuspended in buffer A (50 mM Tris pH 8.0, 500 mM NaCl, 1 mM DTT, 10 mM imidazole and 10% glycerol) and lysed by five passes through an Avestin C5 homogenizer at 15,000 p.s.i. Clarified supernatant was passed over a chelating sepharose fast flow column (GE Healthcare) equilibrated with nickel sulfate and buffer A. Resin was washed with 20 column volumes of buffer A, followed by 20 column volumes of buffer A with 30 mM imidazole. Proteins were eluted with 10 column volumes of buffer A with 300 mM imidazole. The His-MBP tag was removed by overnight dialysis at 4°C with TEV protease in buffer A, and passed back through the nickel affinity column to remove the cleaved tag and protease. Flow through containing untagged Lon was concentrated to 1 mL and run over a S200 10/30 Increase gel filtration column equilibrated with 20 mM Tris pH 8.0, 200 mM NaCl, 1 mM DTT, 25 mM MgCl_2_ and 20% glycerol. Fractions containing Lon were pooled, flash frozen in liquid N_2_ and stored at −80°C until required.

### pINV loss assays

Plasmid loss assays were performed by red-white screening of colonies on solid media containing CR, or on media with sucrose for strains containing s*acB-neo^R^* in *mxiH* (which is located in the T3SS PAI), or using pSTAB2 as described previously (13). For red-white screening, bacteria were grown in liquid culture at 21°C with sub-culturing into fresh LB every 24 h; bacteria were diluted and plated onto solid media containing CR at relevant time points. For *sacB-neo^R^* assays, bacteria retaining pINV or pSTAB2 after ~25 generations of growth on solid media at 21°C or 37°C were detected by serially diluting colonies in PBS and plating onto media selecting for kanamycin-resistant or sucrose-resistant bacteria to detect the presence (pSTAB2^+^ or PAI^+^) or absence (pSTAB2^-^ or PAI^-^) of the *sacB-neo^R^* cassette, respectively. pSTAB2 derivatives containing either the native (p53G_vapBC) or a constitutive (pJ23101) promoter upstream of *vapBC* were used. Plasmids were initially transformed into *E. coli* DH5α and verified by sequencing (Source BioScience) then introduced into *S. sonnei* lacking pINV (GMCT301), or *S. flexneri* BS176 or BS176Δ*lon* by transformation.

### Lon proteolysis assays

*S. sonnei* hexameric Lon (Lon_6_; 166 nM) was combined with 2.5 µM VapBC octamer, 8 mM ATP (Sigma), 10 mM MgCl_2_, and 50 mM Tris pH 8.0 in a total volume of 100 µL. Assays were incubated at 37°C, and samples immediately combined with SDS-PAGE loading dye then incubated at 100°C for 10 min (57). To assess proteolysis in the presence of DNA and polyphosphate, assays were supplemented with 1.5 µM double-stranded DNA (dsDNA) or 1 mM polyphosphate 700 (Kerafast). Promoter DNA (pDNA, 80 bp) was generated by annealing the primer pDNA with a reverse complementary primer; as a control, conDNA (68 bp) was made by annealing the primer conDNA with a reverse complementary primer. Samples were resolved by SDS-PAGE, and the gels were stained with Coomassie blue.

### Mass spectrometry

Intact mass spectrometry was performed as previously described (41). Products of Lon proteolysis assays were diluted 1:50 with 0.1% (v/v) formic acid and reverse-phase chromatography was performed in-line prior to analysis on an MSD-TOF electrospray ionization orthogonal time of flight mass spectrometer (Agilent Technologies Inc.). Following data acquisition, results were evaluated using the MassHunter Qualitative Analysis program (Agilent Technologies). m/z spectra were deconvoluted between minimum and maximum expected masses using the maximum entropy algorithm. Peak masses were compared with the expected average masses. For LC-MS/MS, 100 mM ammonium bicarbonate and 1 µg/mL trypsin (Sigma Aldrich) were added to the products of Lon proteolysis assays and samples incubated overnight at 37°C, prior to analysis on an AmaZon IonTrap (Brucker). Data were analyzed using MASCOT (MatrixScience) and MassHunter Qualitative Analysis data analysis program (Agilent Technologies).

### DNA binding assays

dsDNA for EMSA was generated by annealing equimolar amounts of complementary primers at reducing temperatures from 99°C to 20°C (33 bp with OS1 using SH176 and a reverse complementary primer, or a 68 bp intergenic region with SH159 and a reverse complementary primer), or by amplifying the promoter from *vapBC* (227 bp pVapBC with primers GM415 and GP68) or the constitutive J23101 promoter (150 bp with primers SH136 and GP68) from corresponding versions of pSTAB with primers. Assays were performed as described (9) with VapBC (0 to 352 nM) and 88 nM dsDNA in 20 mM Tris pH 7.5, 100 mM KCl, 2 mM MgCl_2_, 1 mM DTT, 10% glycerol. Reactions were incubated at 37°C for 20 min, resolved on 7% TBE acrylamide gels supplemented with 10% glycerol, stained with SybrSafe and imaged on a GelDoc system.

Surface plasmon resonance was performed on a Biacore 3000 instrument at 25°C. Biotinylated dsDNA (SH167 with SH181) was coupled to streptavidin on the sensor surface (GE Healthcare) at equivalent levels, resulting in a 100 to 150 RU increase. VapBC was then flowed over the chip surface in 50 mM Tris pH 8.0, 150 mM NaCl, and 0.005% (v/v) Tween-20 at a flow rate of 40 µL min^−1^, followed by injection of 10 µL 0.5 M NaCl to remove nonspecific interactions. A control trace was collected using an uncoupled channel. Kinetic fits were performed on data from two separate chips, and two batches of purified VapBC (one starting at 3.2 µM and the other at 2.7 µM). A *K*_D_ estimate for VapBC binding to DNA was taken as the average value after kinetic fits.

Blue–white colony screening of *vapB* tolerance mutations was performed in TB28 (MG1655 Δ*lacZYA*) using pBAD33 expression the different VapBC variants (VapB^T3N^, VapB^T3N+L7P^, VapB^V5E^, VapB^L7P^, VapB^A13P^, VapB^L16R^, VapB^V20G^, and VapB^T3N+A13P+L16R^) and pGH254 expressing LacZ under the *E. coli* native *vapBC* promoter. Overnight cultures were diluted in phosphate-buffered saline (PBS) to OD_600_ = 1 and serially diluted from 10^−1^ to 10^−8^. Serial dilutions were spotted on agar plates containing 50 µg/mL chloramphenicol, 25 µg/mL kanamycin, 40 µg/mL 5-bromo-4-chloro-3-indolyl-β-D-galacto-pyranoside (X-gal) and 0.2% w/v glucose or 0.2% w/v arabinose. Inhibition of the *vapBC* promoter was observed by the presence of white colonies.

### Structural analysis

Plasmids expressing wild-type and mutant VapBC complexes were introduced into *E. coli* BL21(DE3) using the heat shock method for protein expression. Cells were grown in lysogeny broth to OD_600_ ~ 0.6–0.8 and induced using 1 mM IPTG, then incubated at 20°C overnight and harvested by centrifugation at 7,800*×g*. Cells were opened by sonication using a Sonopuls ultrasonic homogenizer (Bandelin) 3 × 5 min at 35% power in Buffer A (500 mM NaCl, 50 mM Hepes pH 7.0, 5 mM MgCl_2_, 5 mM BME) with 20 mM Imidazole. The lysate was cleared by centrifugation at 23,500×*g*, and the supernatant was loaded unto 1 mL HisTrap FF column (Cytiva) equilibrated in Buffer A with 20 mM imidazole, then washed with 10 CV of wash buffers with increasing imidazole (Buffer A with 30-, 50-, 75- and 150 mM imidazole). The protein was eluted using 300 mM imidazole in Buffer A and precipitated using 40% (w/v) ammonium sulfate and stored on ice overnight. Precipitated protein was pelleted and resuspended in Buffer B (50 mM Sodium Citrate pH 5.0, 5 mM MgCl_2_, 5 mM BME) and separated using size exclusion using a Superdex 200 10/300 Gl (Cytiva) equilibrated in Buffer C (500 mM NaCl, 50 mM Hepes pH 5.0, 5 mM MgCl_2_, 5 mM BME). Purified protein complexes were concentrated to a final concentration of 4–7 mg/mL using 50 kDa Vivaspin filters before using Mosquito (STP Labtech) to aliquot 100 nL protein +100 nL reservoir drops in SWISSCI 96-well two-drop MRC crystallization plates. Hits were found in 0.2 M LiSO_4_, 0.1 M sodium citrate pH 5.0–5.5, 26% (v/v) PEG 200. Crystals were cryo-protected in reservoir buffer supplemented with 35-45% (v/v) PEG200 or 20% (v/v) glycerol. Complete X-ray diffraction data sets were collected from single crystals at beamlines P13 and P14 at PETRA III (DESY, Hamburg, Germany) operated by the EMBL and at the BioMax beamline at MAX IV (Lund, Sweden) (58–61). X-ray data reduction and processing were done in XDS (62), after which the structures were determined by molecular replacement using PHASER inside Phenix (63, 64). For wild-type *E. coli* VapBC, the structure of VapBC from *S. flexneri* (PDB: 3TND) was used (9). Refinement was done in phenix.refine (65). For the mutant data sets (VapB^T3N^, VapB^V5E^, and VapB^T3N+A13P+L16R^), the R-free set from wild-type VapBC was transferred and extended using CAD in CCP4 to avoid model bias (66), after which the mutant structures were determined by cross-phasing using the wild-type structure.

### Statistical and computational methods

Data analysis was performed in GraphPad Prism. Data were log-transformed and assessed for normal distribution. Data were then analyzed using one-way or two-way ANOVA with appropriate multiple comparisons tests as indicated in the Figure legends. For non-normal distributed data, Kruskal–Wallis was used for analysis. Relative quantification of Coomassie stained SDS-PAGE gels was performed by visualization on a BioRad Gel Doc XR^+^ using Image Lab software (BioRad).

## Data Availability

All data are available under BioProject accession number PRJNA1098244 and BioSample accession number SAMN40904935. Illumina raw read data and Nanopore base-called data were submitted to SRA and are available via accession numbers SRR28604138 and SRR28604139, respectively. Atomic coordinates and structure factors have been deposited in the Protein Data Bank (PDB) with accession codes 9H6A (VapBC wildtype), 9H6B (VapBC^T3N+A13P+L16R^), 9H6C (VapBC^T3N^), and 9H6D (VapBC^V5E^).

## SUPPLEMENTAL MATERIAL

Supplemental figures mbio.02616-24-s0001.pdf Figures S1–S10.

Supplemental tables and legends mbio.02616-24-s0002.pdf Tables S1–S5 and legends for supplemental figures.

ASM does not own the copyrights to Supplemental Material that may be linked to, or accessed through, an article. The authors have granted ASM a non-exclusive, world-wide license to publish the Supplemental Material files. Please contact the corresponding author directly for reuse.
