# Supplementary material for: Shared mechanisms of enhanced plasmid maintenance and antibiotic tolerance mediated by the VapBC toxin:antitoxin system
Source: mBio. 2024 Dec 20;16(2):e02616-24. doi: 10.1128/mbio.02616-24 (PMC11796401; doi:10.1128/mbio.02616-24)

Supplementary Figure 1.

```
53G   GATACTCATCATAAACGTATATCCCTTTGACATATCCCGGTATCAATCCCACAATAGATA 60
CS14  GATACTCATCATAAACGTATATCCCTTTGACATATCCCGGCATCAATCCCACAATAGATA 60
                          O1  -35                    -10  O2

53G   TACACAAGACATATCCACATAAGGAGGCAAATAATGGAAACCACCGTATTTCTCAGCAAC 120
CS14  TACACAAGACATATCCACATAAGGAGGCAAATAATGGAAACCACCGTATTTCTCAGCAAC 120
           O2                             M  E  T  T  V  F  L  S  N

53G   CGCAGCCAGGCGGTCAGACTGCCAAAAGCGGTTGCATTGCCGGAAAACGTAAAACGCGTT 180
CS14  CGCAGCCTGGCGGTCAGACTGCCAAAAGCGGTTGCATTGCCGGAAAACGTAAAACGCGTT 180
       R  S  L  A  V  R  L  P  K  A  V  A  L  P  E  N  V  K  R  V

53G   GAAGTGATTGCTGTCGGACGAACCAGAATCATTACGCCAGCCGGAGAGACATGGGACGAA 240
CS14  GAAGTGATTGCTGTCGGACGAACCAGAATCATTACGCCAGCCGGAGAGACATGGGACGAA 240
       E  V  I  A  V  G  R  T  R  I  I  T  P  A  G  E  T  W  D  E

53G   TGGTTCGACGGACACAGCGTCAGCGCCGATTTTATGGATAACAGGGAACAGCCCGGCATG 300
CS14  TGGTTCGACGGACACAGCGTCAGCGCCGATTTTATGGATAACAGGGAACAGCCCGGCATG 300
       W  F  D  G  H  S  V  S  A  D  F  M  D  N  R  E  Q  P  G  M

53G   CAGGAAAGGGAGTCATTCTGATGCTGAAGTTTATGCTCGATACCAACATCTGCATTTTTA 360
CS14  CAGGAAAGGGAGTCATTCTGATGCTGAAGTTTATGCTCGATACCAACATCTGCATTTTTA 360
       Q  E  R  E  S  F  *  M  L  K  F  M  L  D  T  N  I  C  I  F

53G   CGATAAAGAACAAACCCGCCAGCGTCAGGGAGCGTTTTAACCTGAACCAGGGGAGAATGT 420
CS14  CGATAAAGAACAAACCCGCCAGCGTCAGGGAGCGTTTTAACCTGAACCAGGGGAGAATGT 420
       T  I  K  N  K  P  A  S  V  R  E  R  F  N  L  N  Q  G  R  M

53G   GTATCAGTTCGGTCACCCTGATGGAGCTGATATATGGTGCAGAAAAAAGCCAGATGCCTG 480
CS14  GTATCAGTTCGGTCACCCTGATGGAGCTGATATATGGTGCAGAAAAAAGCCAGATGCCTG 480
       C  I  S  S  V  T  L  M  E  L  I  Y  G  A  E  K  S  Q  M  P

53G   AACGTAATCTCGCTGTGATCGAGGGATTTGTTTCCCGCATTGATGTTCTGGATTACGACG 540
CS14  AACGTAATCTCGCTGTGATCGAGGGATTTGTTTCCCGCATTGATGTTCTGGATTACGACG 540
       E  R  N  L  A  V  I  E  G  F  V  S  R  I  D  V  L  D  Y  D

53G   CTGCTGCAGCCACACACACCGGCCAGATAAGAGCAGAACTTGCCCGTCAGGGACGCCCTG 600
CS14  CTGCTGCAGCCACACACACCGGCCAGATAAGAGCAGAACTTGCCCGTCAGGGACGCCCTG 600
       A  A  A  A  T  H  T  G  Q  I  R  A  E  L  A  R  Q  G  R  P

53G   TCGGGCCATTTGATCAAATGATCGCAGGTCATGCCCGCAGTCGGGGGCTGATTATTGTGA 660
CS14  TCGGGCCATTTGATCAAATGATCGCAGGTCATGCCCGCAGTCGGGGGCTGATTATTGTGA 660
       V  G  P  F  D  Q  M  I  A  G  H  A  R  S  R  G  L  I  I  V

53G   CTAATAACACCCGGGAATTTGAACGTGTGGGCGGCCTGAGAACTGAAGACTGGAGCTGA  719
CS14  CTAATAACACCCGGGAATTTGAACGTGTGGGCGGCCTGAGAACTGAAGACTGGAGCTGA  719
       T  N  N  T  R  E  F  E  R  V  G  G  L  R  T  E  D  W  S  *
```

Supplementary Figure 2.

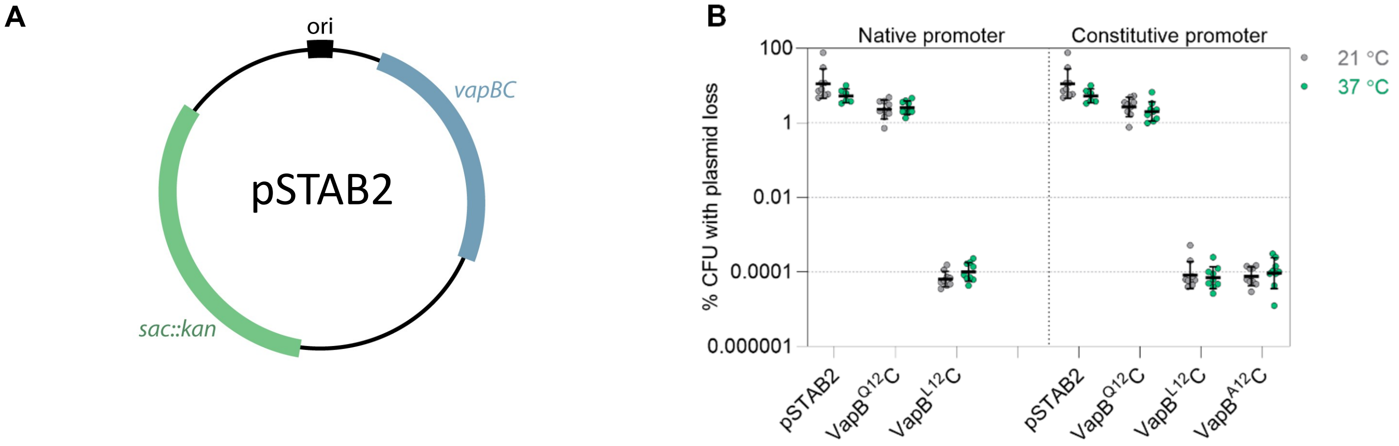

Supplementary Figure 3.

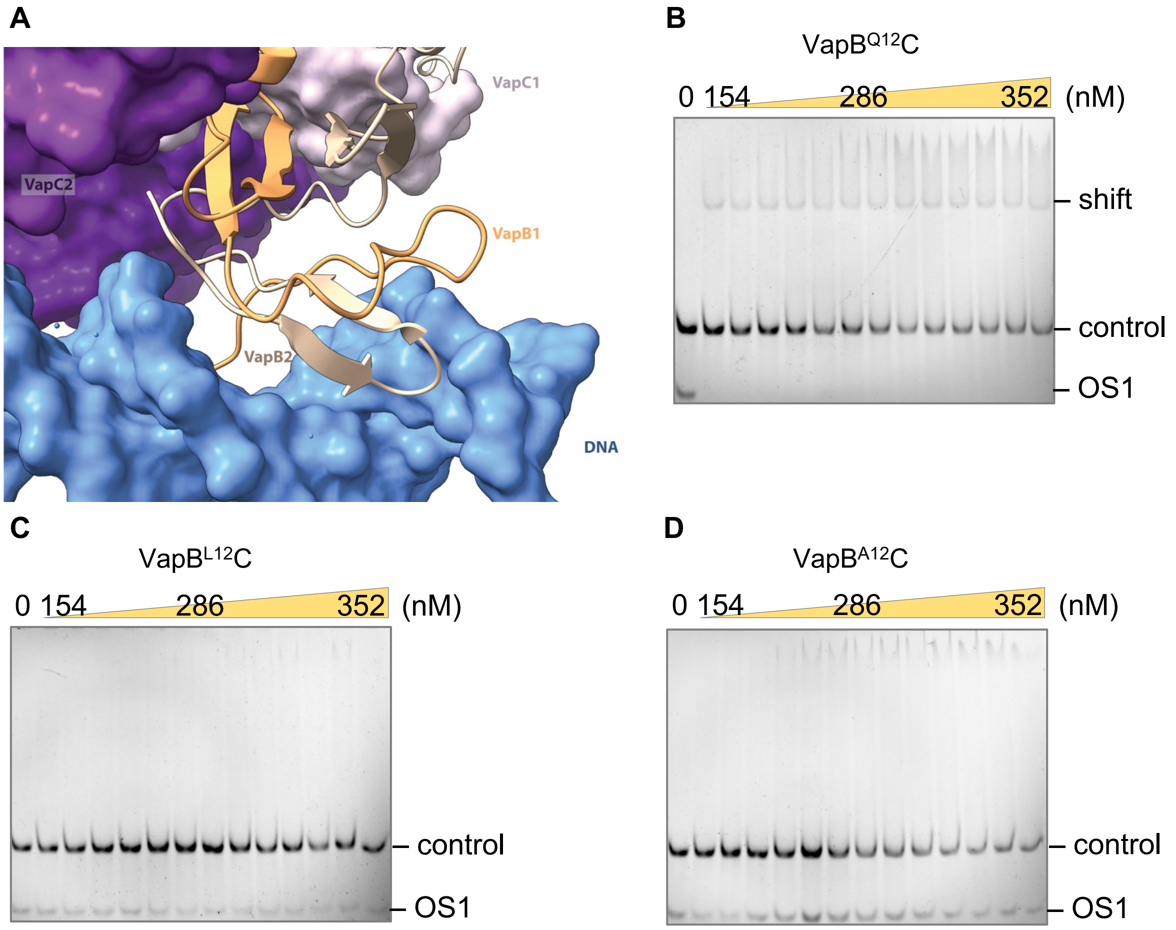

Supplementary Figure 4.

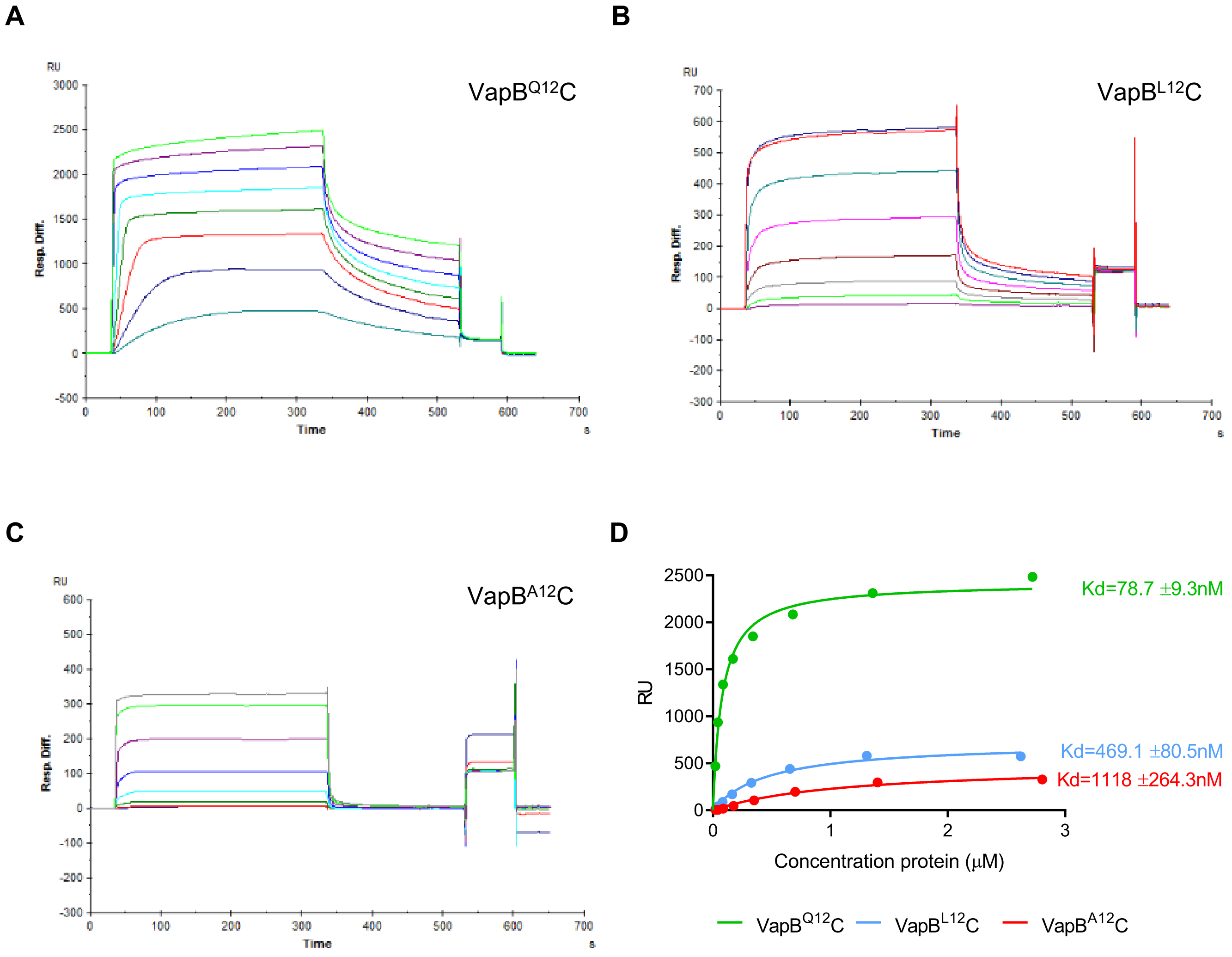

Supplementary Figure 5.

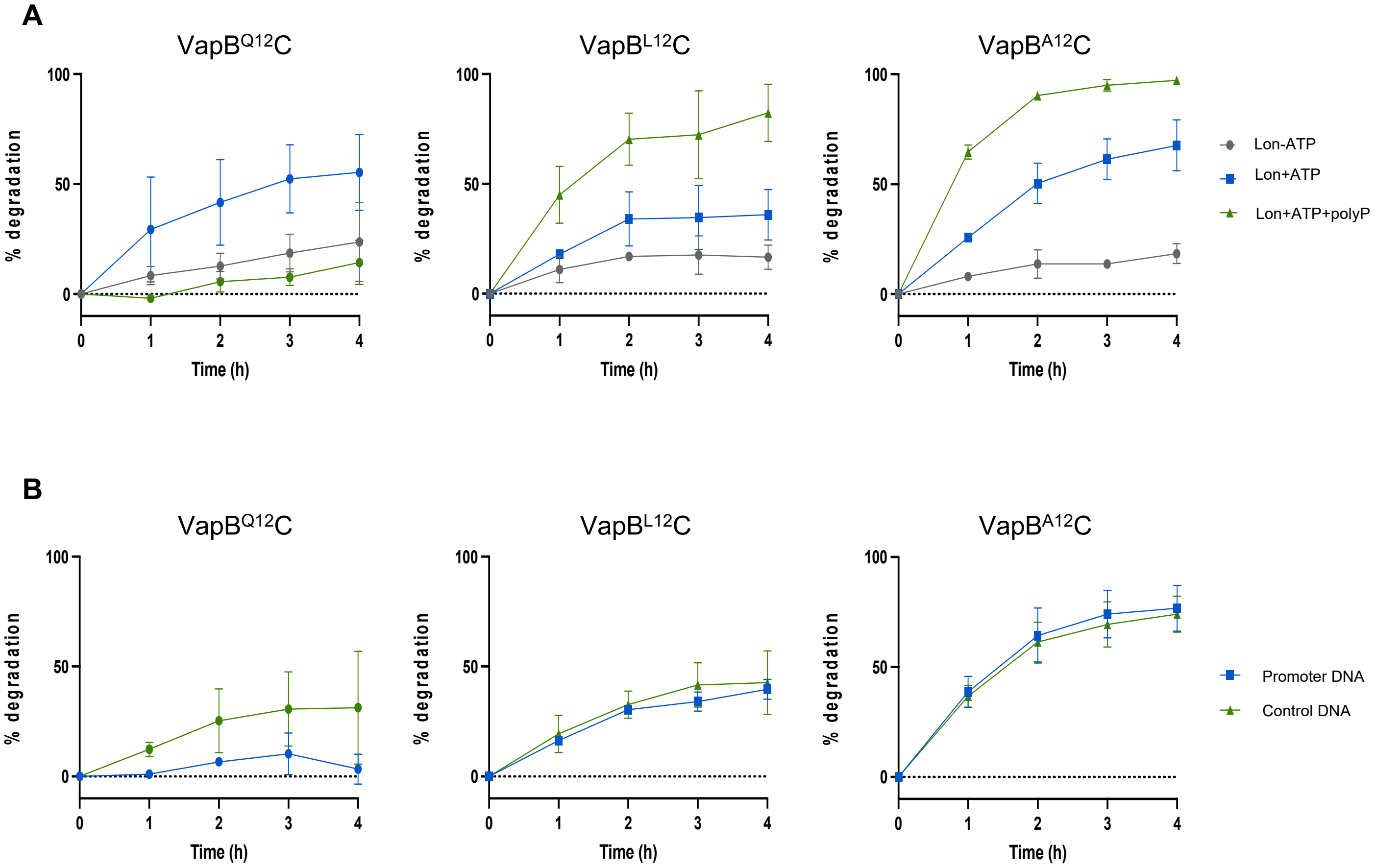

Supplementary Figure 6.

A

VapB^{12Q}C tryptic fragments
METTVFLSNRSQAVRLPKAVALPENVKRVEVIAVGRTRIITPAGETWDEWFDGHSVSADFMDNREQPGMQERESF
METTVFLSNRSQAVRLPKAVALPENVKRVEVIAVGRTRIITPAGETWDEWFDGHSVSADFMDNREQPGMQERESF
METTVFLSNRSQAVRLPKAVALPENVKRVEVIAVGRTRIITPAGETWDEWFDGHSVSADFMDNREQPGMQERESF

VapB^{12Q}C+Lon tryptic fragments
METTVFLSNRSQAVRLPKAVALPENVKRVEVIAVGRTRIITPAGETWDEWFDGHSVSADFMDNREQPGMQERESF
METTVFLSNRSQAVRLPKAVALPENVKRVEVIAVGRTRIITPAGETWDEWFDGHSVSADFMDNREQPGMQERESF
METTVFLSNRSQAVRLPKAVALPENVKRVEVIAVGRTRIITPAGETWDEWFDGHSVSADFMDNREQPGMQERESF

VapB^{12Q}C+Lon+Pp tryptic fragments
METTVFLSNRSQAVRLPKAVALPENVKRVEVIAVGRTRIITPAGETWDEWFDGHSVSADFMDNREQPGMQERESF
METTVFLSNRSQAVRLPKAVALPENVKRVEVIAVGRTRIITPAGETWDEWFDGHSVSADFMDNREQPGMQERESF
METTVFLSNRSQAVRLPKAVALPENVKRVEVIAVGRTRIITPAGETWDEWFDGHSVSADFMDNREQPGMQERESF
METTVFLSNRSQAVRLPKAVALPENVKRVEVIAVGRTRIITPAGETWDEWFDGHSVSADFMDNREQPGMQERESF

B

VapB^{12L}C tryptic fragments
METTVFLSNRSLAVRLPKAVALPENVKRVEVIAVGRTRIITPAGETWDEWFDGHSVSADFMDNREQPGMQERESF
METTVFLSNRSLAVRLPKAVALPENVKRVEVIAVGRTRIITPAGETWDEWFDGHSVSADFMDNREQPGMQERESF
METTVFLSNRSLAVRLPKAVALPENVKRVEVIAVGRTRIITPAGETWDEWFDGHSVSADFMDNREQPGMQERESF

VapB^{12L}C+Lon tryptic fragments
METTVFLSNRSLAVRLPKAVALPENVKRVEVIAVGRTRIITPAGETWDEWFDGHSVSADFMDNREQPGMQERESF
METTVFLSNRSLAVRLPKAVALPENVKRVEVIAVGRTRIITPAGETWDEWFDGHSVSADFMDNREQPGMQERESF
METTVFLSNRSLAVRLPKAVALPENVKRVEVIAVGRTRIITPAGETWDEWFDGHSVSADFMDNREQPGMQERESF

VapB^{12L}C+Lon Pp tryptic fragments
METTVFLSNRSLAVRLPKAVALPENVKRVEVIAVGRTRIITPAGETWDEWFDGHSVSADFMDNREQPGMQERESF
METTVFLSNRSLAVRLPKAVALPENVKRVEVIAVGRTRIITPAGETWDEWFDGHSVSADFMDNREQPGMQERESF
METTVFLSNRSLAVRLPKAVALPENVKRVEVIAVGRTRIITPAGETWDEWFDGHSVSADFMDNREQPGMQERESF
METTVFLSNRSLAVRLPKAVALPENVKRVEVIAVGRTRIITPAGETWDEWFDGHSVSADFMDNREQPGMQERESF

C

VapB^{12A}C tryptic fragments
METTVFLSNRSAAVRLPKAVALPENVKRVEVIAVGRTRIITPAGETWDEWFDGHSVSADFMDNREQPGMQERESF
METTVFLSNRSAAVRLPKAVALPENVKRVEVIAVGRTRIITPAGETWDEWFDGHSVSADFMDNREQPGMQERESF
METTVFLSNRSAAVRLPKAVALPENVKRVEVIAVGRTRIITPAGETWDEWFDGHSVSADFMDNREQPGMQERESF

VapB^{12A}C+Lon tryptic fragments
METTVFLSNRSAAVRLPKAVALPENVKRVEVIAVGRTRIITPAGETWDEWFDGHSVSADFMDNREQPGMQERESF
METTVFLSNRSAAVRLPKAVALPENVKRVEVIAVGRTRIITPAGETWDEWFDGHSVSADFMDNREQPGMQERESF
METTVFLSNRSAAVRLPKAVALPENVKRVEVIAVGRTRIITPAGETWDEWFDGHSVSADFMDNREQPGMQERESF

VapB^{12A}C+Lon+Pp tryptic fragments
METTVFLSNRSAAVRLPKAVALPENVKRVEVIAVGRTRIITPAGETWDEWFDGHSVSADFMDNREQPGMQERESF
METTVFLSNRSAAVRLPKAVALPENVKRVEVIAVGRTRIITPAGETWDEWFDGHSVSADFMDNREQPGMQERESF
METTVFLSNRSAAVRLPKAVALPENVKRVEVIAVGRTRIITPAGETWDEWFDGHSVSADFMDNREQPGMQERESF
METTVFLSNRSAAVRLPKAVALPENVKRVEVIAVGRTRIITPAGETWDEWFDGHSVSADFMDNREQPGMQERESF

Supplementary Figure 7.

Peptide: MDNREQPGMQERESF

Neutral mass	M+H+	M+2H2+	M+3H3+
1853	1854	927	618

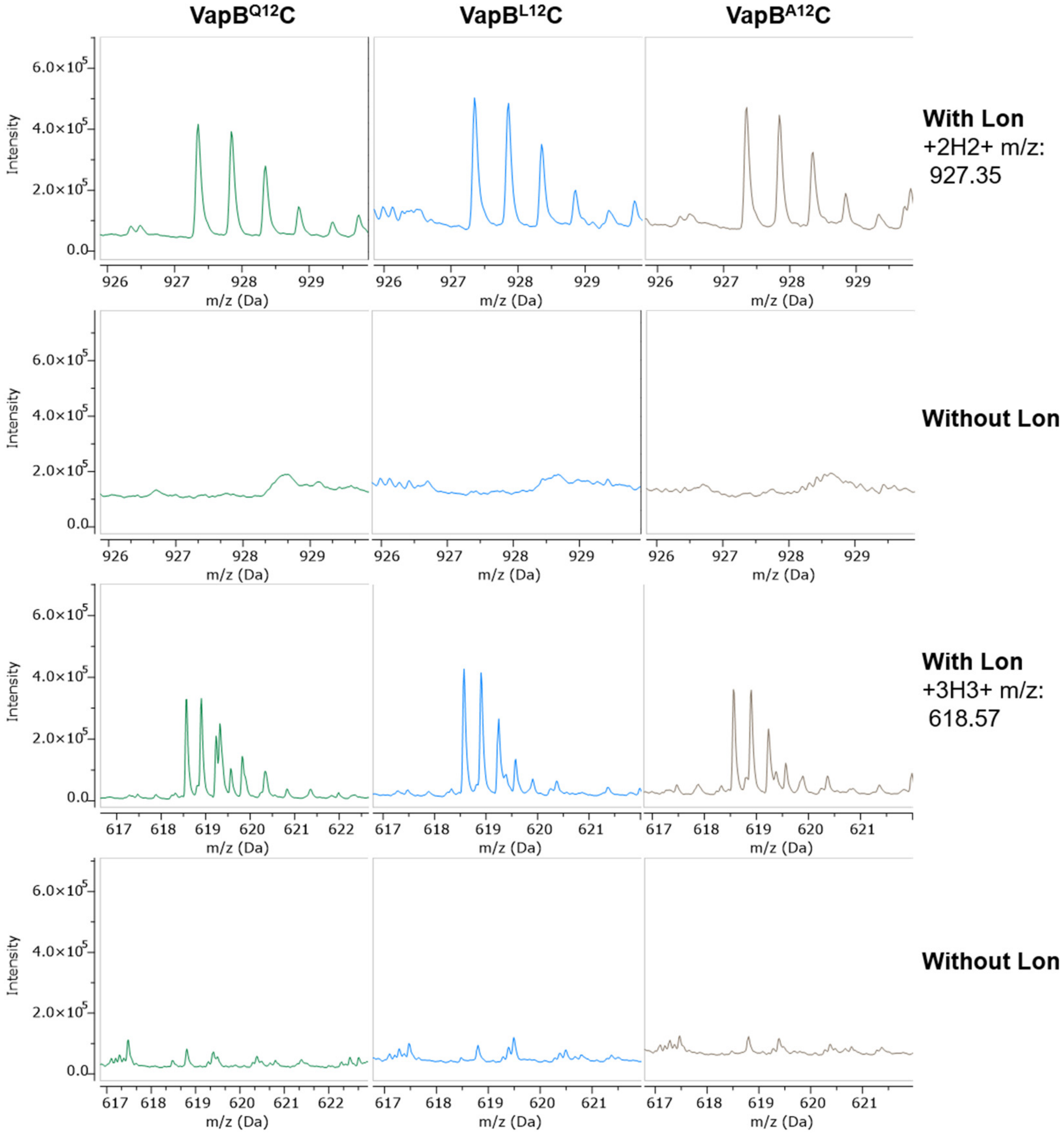

Supplementary Figure 8.

Peptide: DGHSVSADFMDNREQPGMQERESF

Neutral mass	M+H+	M+2H2+	M+3H3+
2768	2769	1385	923

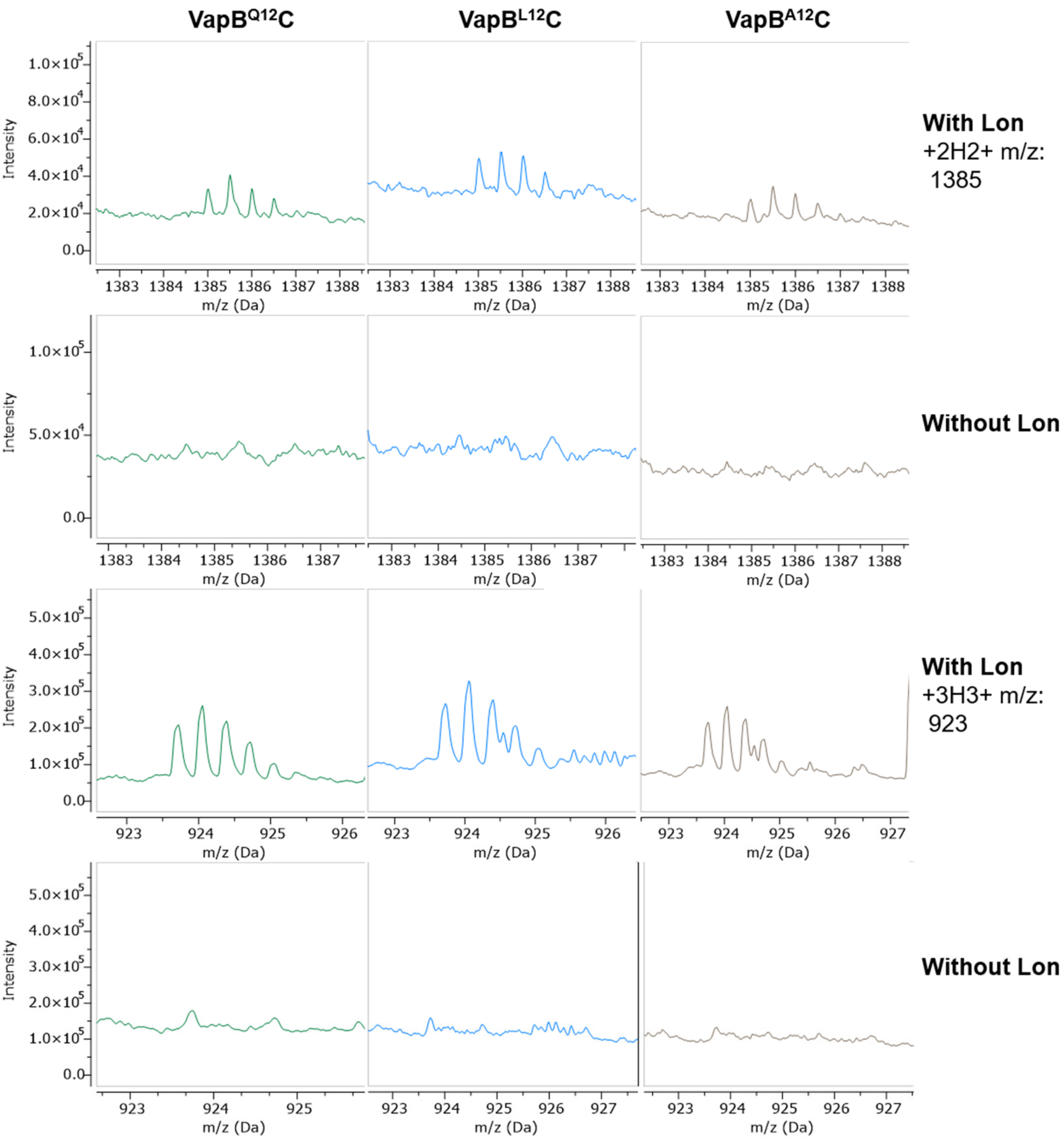

Supplementary Figure 9.

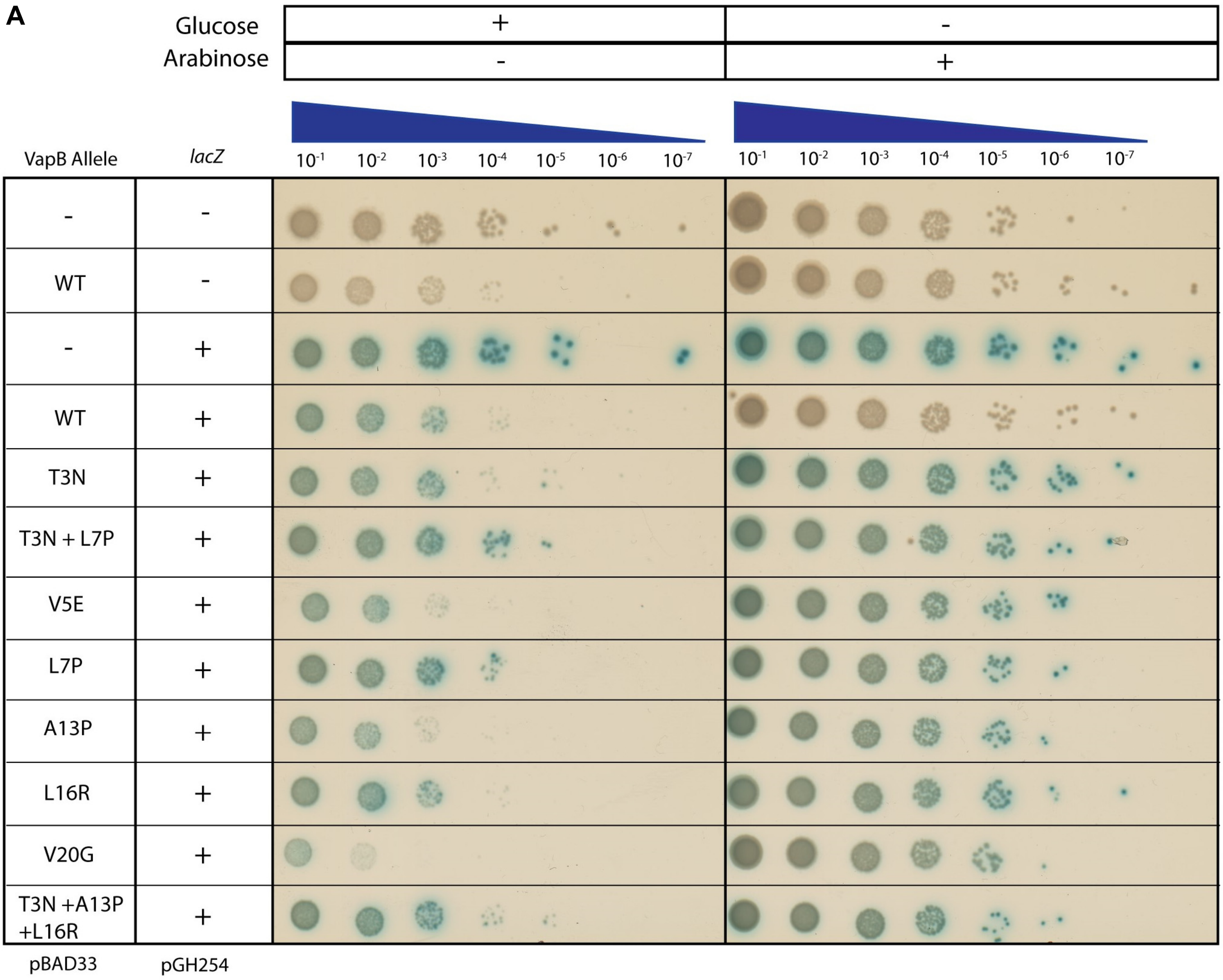

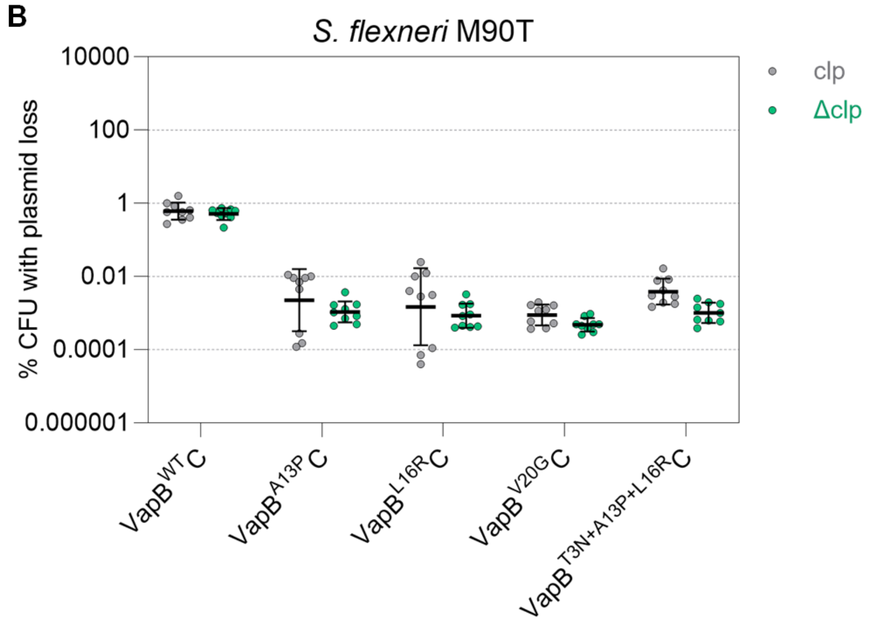

Supplementary Figure 10.

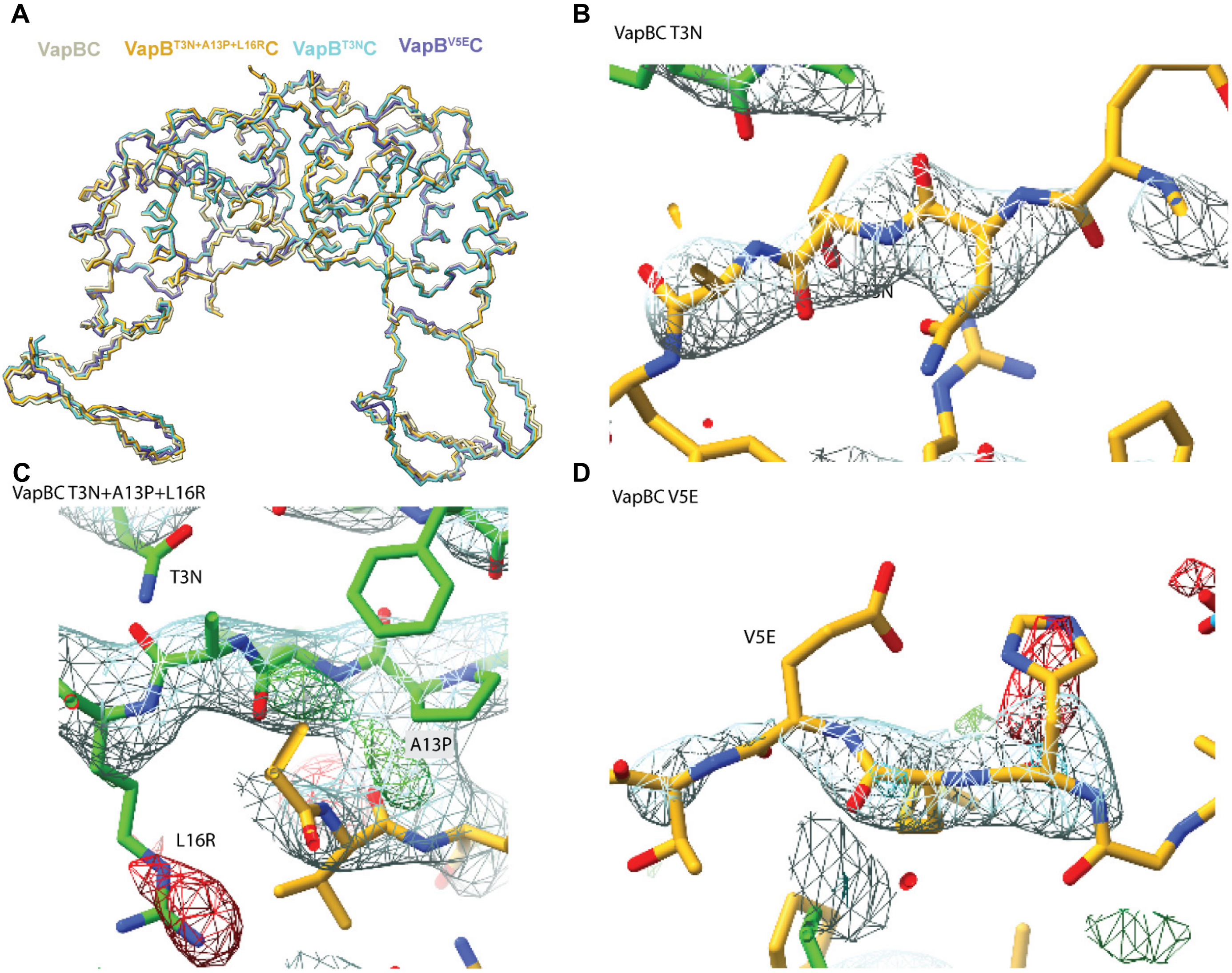

Supplement: Supplemental figures — Figures S1–S10. [file mbio.02616-24-s0001.pdf]
