# Supplementary material for: Shared mechanisms of enhanced plasmid maintenance and antibiotic tolerance mediated by the VapBC toxin:antitoxin system
Source: mBio. 2024 Dec 20;16(2):e02616-24. doi: 10.1128/mbio.02616-24 (PMC11796401; doi:10.1128/mbio.02616-24)
Supplement: Supplemental tables and legends — Tables S1–S5 and legends for supplemental figures. [file mbio.02616-24-s0002.pdf]

Supplementary Table 1. Bacterial strains used in this study

Strain	Relevant genotype/ description	Reference
S. sonnei 53G	Wild-type *S. sonnei* 53G	[75]
S. flexneri M90T	Wild-type *S. flexneri* serotype 5a	[76]
BS176	*S. flexneri* lacking pINV	[76]
S. sonnei pINV	*S. sonnei* lacking pINV	[19]
S. sonnei CS14	Wild-type *S. sonnei* CS14	[36]
E. coli MG1655	Wild-type *E. coli* K-12	[77]
E. coli TB28	*E. coli* K-12 Δ*lacZYA*	[78]
E. coli C41	*ompT hsdSB* (*rB- mB-*) *gal dcm* (DE3)	[79]
E. coli DH5α	*fhuA*2 Δ(*argF-lacZ*) *U169 phoA glnV44* Φ*80* Δ(*lacZ*)*M15 gyrA96 recA1 relA1 endA1 thi-1 hsdR17*	[80]
E. coli B834	F^- *ompT hsdS*$_B$(r_B^- m_B^-) *gal dcm met* (DE3)	[81]
S. flexneri M90T Δ*lon*	Δ*lon*	This work
S. flexneri M90T Δ*clpP*	Δ*clpP*	This work
GMCT307 & GMCT311	*S. sonnei* 53G *mxiH::sacB-neo*^{R} VapB^{Q12}-*cat* (two independently clones)	This work
GMCT309 & GMCT312	*S. sonnei* 53G *mxiH::sacB-neo*^{R} VapB^{L12}-*cat* (two independently-created clones)	This work

GMCT332 & GMCT333	*S. sonnei* CS14 *mxiH::sacB-neo*^{R} VapB^{L12}-*cat* (two independently-created clones)	This work
GMCT334 & GMCT335	*S. sonnei* CS14 *mxiH::sacB-neo*^{R} VapB^{Q12}-*cat* (two independently-created clones)	This work

Supplementary Table 2. Plasmids used in this study

Plasmid	Description	Reference
pET28a-VapCB		This work
pET28a-VapCB^{L12}	Expression of His-VapC and VapB$^{Q12/L12/A12}$	
pET28a-VapCB^{A12}		
pET28a-MBP-Lon	For expression of His-MBP-Lon	This work
pSTAB2	6.3 kb plasmid containing the pINV ori and *sacB-neo*	[5]
pSTAB2-VapBC		This work
pSTAB2-VapB^{A12}C	pSTAB with *S. sonnei* VapB$^{Q12/L12/A12}$C	
pSTAB2-VapB^{A12}C		
pSTAB2-VapB^{F6A}C		This work
pSTAB2-VapB^{F51A}C	pSTAB with *S. sonnei* VapB$^{F6A/F51A/F60A}$C	
pSTAB2-VapB^{F60A}C		
pUC19-Lon:cam^{R}	For disruption of Lon protease with cam cassette	[5]
pCP20	FLP recombinase for λ Red cassette excision	[56]
pKD3	Source of *camR* cassette for λ Red recombination	[82]
pKD46	Helper plasmid for λ Red recombination	[82]

pUC19	High-copy cloning vector	PMID 2985470
pGM195	pUC19 with VapB^{Q12}-*cat* and 53G flanking sequence for creation of GMCT307 & GMCT311	This study
pGM197	pUC19 with VapB^{L12}-*cat* and 53G flanking sequence for creation of GMCT309 & GMCT312	This study
pGM203	pUC19 with VapB^{L12}-*cat* and CS14 flanking sequence for creation of GMCT332 & GMCT333	This study
pGM205	pUC19 with VapB^{Q12}-*cat* and CS14 flanking sequence for creation of GMCT334 & GMCT335	This study
pET28a-VapBC	Expression of His-TEV-VapB and VapC	This work
pET28a-VapB^{T3N}C	Expression of His-TEV-VapB^{T3N} and VapC	This work
pET28a-VapB^{V5E}C	Expression of His-TEV-VapB^{V5E} and VapC	This work
pET28a-VapB$^{T3N+A13P+L16R}$	Expression of His-TEV-VapB$^{T3N+A13P+L16R}$ and VapC	This work
pSTAB2-VapB^{T3N}C	pSTAB with *S. sonnei* VapB^{T3N}C under native or constitutive (J23101) promoter	This work
pSTAB2-VapB$^{T3N+L7P}$C	pSTAB with *S. sonnei* VapB^{T3N}C under native or constitutive (J23101) promoter	This work
pSTAB2-VapB^{V5E}C	pSTAB with *S. sonnei* VapB$^{T3N+L7P}$C under native or constitutive (J23101) promoter	This work
pSTAB2-VapB^{L7P}C	pSTAB with *S. sonnei* VapB^{L7P}C under native or constitutive (J23101) promoter	This work
pSTAB2-VapB^{A13P}C	pSTAB with *S. sonnei* VapB^{A13P}C under native or constitutive (J23101) promoter	This work
pSTAB2-VapB^{L16R}C	pSTAB with *S. sonnei* VapB^{L16R}C under native or constitutive (J23101) promoter	This work
pSTAB2-VapB^{V20G}C	pSTAB with *S. sonnei* VapB^{V20G}C under native or constitutive (J23101) promoter	This work

pSTAB2-VapB$^{T3N+A13P+L16R}$C	pSTAB with *S. sonnei* VapB$^{T3N+A13P+L16R}$C under native or constitutive (J23101) promoter	This work
pBAD33-VapBC	Expression of *vapB* and *vapC* each with optimized SD under a single promoter induced by arabinose or inhibited by glucose.	This work
pBAD33-VapB^{T3N}C	Expression of *vapB*^{T3N} and *vapC* each with optimized SD under a single promoter induced by arabinose or inhibited by glucose.	This work
pBAD33-VapB$^{T3N+L7P}$C	Expression of *vapB*$^{T3N+L7P}$ and *vapC* each with optimized SD under a single promoter induced by arabinose or inhibited by glucose.	This work
pBAD33-VapB^{V5E}C	Expression of *vapB*^{V5E} and *vapC* each with optimized SD under a single promoter induced by arabinose or inhibited by glucose.	This work
pBAD33-VapB^{L7P}C	Expression of *vapB*^{L7P} and *vapC* each with optimized SD under a single promoter induced by arabinose or inhibited by glucose.	This work
pBAD33-VapB^{A13P}C	Expression of *vapB*^{A13P} and *vapC* each with optimized SD under a single promoter induced by arabinose or inhibited by glucose.	This work
pBAD33-VapB^{L16R}C	Expression of *vapB*^{L16R} and *vapC* each with optimized SD under a single promoter induced by arabinose or inhibited by glucose.	This work
pBAD33-VapB^{V20G}C	Expression of *vapB*^{V20G} and *vapC* each with optimized SD under a single promoter induced by arabinose or inhibited by glucose.	This work
pBAD33-VapB$^{T3N+A13P+L16R}$C	Expression of *vapB*$^{T3N+A13P+L16R}$ and *vapC* each with optimized SD under a single promoter induced by arabinose or inhibited by glucose.	This work
pGH254-nat_*VapBC*	Expression of *LacZYA* under the *E. coli* native *vapBC* promoter.	This work

Supplementary Table 3. Primers used in this study

Primer	Sequence (5′ – 3′)	Vector constructed
SH10	CTGGTGCCGCGCGGCAGCCATATGCTGAAGTTTATGCTCG	pET28a-VapCB
SH11	CCATTATTTGCCTCCTTATCAGCTCCAGTCTTCAGTTC	
SH12	CTGGAGCTGATAAGGAGGCAAATAATGGAAACCACCGTATTTC	
SH13	AGTGGTGGTGGTGGTGGTGCTCGAGTCAGAATGACTCCCTTTC	
	CAGCAACCGCAGCCTGGCGGTCAGACTGCCA	pET28a-VapCB^{L12}
	GTCTGACCGCCAGGCTGCGGTTGCTGAGAAATAC	
SH134	GTCTGACCGCCGCGCTGCGGTTGCTGAGAAATAC	pET28a-VapCB^{A12}
SH135	CAGCAACCGCAGCGCGGCGGTCAGACTGCCA	
SH24	CTTGCGGCCGCATGAATCCTGAGCGTTCTGA	pET28a-MBP-Lon
SH25	GTGCTCGAGCTATTTTGCAGTCACAACCTG	
GM415	CCATATGGCTAGCATGAGCGGATCCGTAATTATCCTTGTCGGTATAATA	pSTAB2-VapBC
GM394	GGTGCTCGAGTGCGGCCGCAAGCTTGAACAGGTCAGCTCCAG	
GP65	AACCGCAGCGCGGCGGTCAGACTGCCAAAA	pSTAB2-VapB^{A12}C
GP66	TCTGACCGCCGCGCTGCGGTTGCTGAGAAATA	

GP67	AACCGCAGCCAGGCGGTCAGACTGCCAAAA	pSTAB2-VapB^{L12}C
GP68	TCTGACCGCCTGGCTGCGGTTGCTGAGAAATA	
SH101	GTTGCTGAGAGCTACGGTGGTTTCCATTATT	pSTAB2-VapB^{F6A}C
SH102	AACCACCGTAGCTCTCAGCAACCGCAGC	
SH103	GTGTCCGTCGGCCCATTCGTCCCATGTCTCTC	pSTAB2-VapB^{F51A}C
SH104	GGACGAATGGGCCGACGGACACAGCGTCAGCG	
SH105	GTTATCCATAGCATCGGCGCTGACGCTGTGTC	pSTAB2-VapB^{F60A}C
SH106	CAGCGCCGATGCTATGGATAACAGGGAACAGC	
SH136	CCATATGGCTAGCATGAGCGGATCCTTTACAGCTAGCTCAGTCCTAGGTATTATGCTA GCACCCGTTTTTTTGGGCTAGC	pSTAB2-J23101-VapBC
SH137	CCATATGGCTAGCATGAGCGGATCCTTTACAGCTAGCTCAGTCCTAGGTATTATGCTA GCACCCGTTTTTTTGGGCTAGC	
SH107	GTAAAACGACGGCCAGTGTTTGAATCGGAAATCGATCTGCT	pCONJ4-lon^{K362Q}
SH108	TGGACTGACCAAGAGAGGTTTGACCTACCCCCGGCGGCCCTAC	
SH109	GTAGGGCCGCCGGGGGTAGGTCAAACCTCTCTTGGTC	
SH110	GCAGGAAACAGCTATGACGTTTATAATGGCGCTATCGTCG	
SH159	TATTGGCTCCTTCTGATGCCATTCTATTTCAGGAAGGAGTGTCCGTTAAACTCAGGCT ACCTCAGACT	EMSA: control DNA

SH176	ATGTGGATATGTCTTGTGTATATCTATTGTGGG	EMSA: OS1
GP007	ACGGCCAGTGAATTCGAGCTCGTGAAGCGGGTCCGGGTG	Upstream sequence for integration of *vapB* mutations
GP010	CCATGGCTAATTCCCATTCAGCTCCAGTCTTCAGTTCTCAG	
GM174	TGTGTAGGCTGGAGCTGCTT	*cat* cassette from pKD3
GM175	ATGGGAATTAGCCATGGTCC	
GM292	CAGCTCCAGCCTACACACCTGTTCATCAGAAATCATCTCC	Downstream sequence for integration of *vapB* mutations
GM293	CTATGACCATGATTACGCCAAGCTTGGAAGCCCGTAAGTTCGG	
GM294	GGCAGTCTGACCGCC**A**GGCTGCGGTTGCTGAGAAATAC	Mutagenic primers for *vapB*^{L12} construction
GM295	CTCAGCAACCGCAGCC**T**GGCGGTCAGACTGCCAAAAGCG	
SH180	ATCATAAACGTATATCCCTTTGACATATCCCG	Biotinylated primers
SH181	CCCACAATAGATATACACAAGACATATCCACAT	for SPR
pDNA	TCATCATAAACGTATATCCCTTTGACATATCCCGGTATCAATCCCACAATAGATATAC ACAAGACATATCCACATAAGGA	*vapBC* promoter for Lon assays
conDNA	AGTCTGAGGTAGCCTGAGTTTAACGGACACTCCTTCCTGAAATAGAATGGCATCAGA AGGAGCCAATA	control DNA for Lon assays
MRN1	CATATCCACATAAGGAGGCAAATAATGGAAAACACCGTATTTCTCAGCAA	pSTAB2-VapB^{T3N}C/ pSTAB2-VapB$^{T3N+A13P+L16R}$C

MRN2	CCATTATTTGCCTCCTTATGTGG	pSTAB2-VapB^{T3N}C/ pSTAB2-VapB$^{T3N+A13P+L16R}$C
MRN3	CACATAAGGAGGCAAATAATGGAAAACACCGTATTTCCCAGCAACCGCAG	pSTAB2-VapB$^{T3N+L7P}$C
MRN4	TTTTCCATTATTTGCCTCCTTATGTG	
MRN5	CACATAAGGAGGCAAATAATGGAAACCACCGTATTTCCCAGCAACCGCAG	pSTAB2-VapB^{L7P}C
MRN6	GGTGGTTTCCATTATTTGCCTCC	
MRN7	CCGTATTTCTCAGCAACCGCAGCCAGCCGGTCAGACTGCCAAAAGCGGTT	pSTAB2-VapB^{A13P}C
MRN8	CTGCGGTTGCTGAGAAATACGG	pSTAB2-VapB^{A13P}C
MRN9	CCGTATTTCTCAGCAACCGCAGCCAGGCGGTCAGACGGCCAAAAGCGGTT	pSTAB2-VapB^{L16R}C
MRN10	CTGCGGTTGCTGAGAAATACGG	pSTAB2-VapB^{L16R}C
MRN11	GCCAGGCGGTCAGACTGCCAAAAGCGGGTGCATTG	pSTAB2-VapB^{V20G}C
MRN12	GGCAATGCACCCGCTTTTG	pSTAB2-VapB^{V20G}C
MRN13	GTTTTCCGGCAATGCACCCG	pSTAB2-VapB^{V20G}C
MRN16	AATTCGAGCTCAAGGAGGCAAATAATGGAAAACACCGTATTTCTCAGCAA	pSTAB2-VapB^{T3N}C
MRN17	TTCCATTATTTGCCTCCTT	pSTAB2-VapB$^{T3N/L7P/T3N+L7P}$C
MRN18	AGCTCAAGGAGGCAAATAATGGAAACCACCGAATTTCTCAGCAACCGCAG	pSTAB2-VapB^{V5E}C
MRN19	AGCTCAAGGAGGCAAATAATGGAAAACACCGTATTTCCCAGCAACCGCAG	pSTAB2-VapB$^{T3N+L7P}$C

MRN20	AGCTCAAGGAGGCAAATAATGGAAACCACCGTATTTCCCAGCAACCGCAG	pSTAB2-VapB^{L7P}C
MRN21	CCGTATTTCTCAGCAACCGCAGCCAGCCGGTCAG	pSTAB2-VapB$^{A13P/T3N+A13P+L16R}$C
MRN22	CTGGCTGCGGTTGCTGAGAAATAC	pSTAB2-VapB$^{A13P/T3N+A13P+L16R}$C
MRN23	CAGCCAGGCGGTCAGACGGCCAAAAGCGGTTG	pSTAB2-VapB$^{L16R/T3N+A13P+L16R}$C
MRN24	TTTGGCCGTCTGACCGC	pSTAB2-VapB$^{A13P/L16R/T3N+A13P+L16R}$C
SHJ1	CCCCCGAATTCCCAGAAGACGGGTAAAAACATC	pGH254-nat_*vapBC*
SHJ2	CATAGGTACCGCAATGCAACCGCTTTTG	
SHJ3	CCCCCGAGCTCTAAGGAGGAAATTAAATGGAAACCACCGTATTTCTC	pBAD33-VapBC, pBAD33-VapB^{A13P}C, pBAD33-VapB^{L16R}C, pBAD33-VapB^{V20G}C, pBAD33-VapB$^{T3N+A13P+L16R}$C
SHJ4	CCCCCGAGCTCTAAGGAGGAAATTAAATGGAAAACACCGTATTTCTCAG	pBAD33-VapB^{T3N}C
SHJ5	CCCCCGAGCTCTAAGGAGGAAATTAAATGGAAAACACCGTATTTCCCAG	pBAD33-VapB$^{T3N+L7P}$C
SHJ6	CCCCCGAGCTCTAAGGAGGAAATTAAATGGAAACCACCGTATTTCCCAG	pBAD33-VapB^{L7P}C
SHJ7	CCCCCGAGCTCTAAGGAGGAAATTAAATGGAAACCACCGAATTTCTCAG	pBAD33-VapB^{V5E}C
MRN25	CCCATGTCGACTCAACTCCAGTCTTCAATTCTCAGG	All above pBAD33

Supplementary Table 4. Crystallographic data statistics

**Structure**	**VapBC^{WT}**	**VapBC$^{T3N+A13P+L16R}$**	**VapBC^{T3N}**	**VapBC^{V5E}**
PDB ID	9H6A	9H6B	9H6C	9H6D
Resolution range (Å)	47.62-2.86 (2.96-2.86)	46.5-2.80 (2.90-2.80)	44.5-2.65 (2.75-2.65)	46.8-3.1 (3.21-3.1)
Space group	P 3_2 2 1	P 3_2 2 1	P 3_2 2 1	P 3_2 2 1
Unit cell (a, b, c) (Å)	90.54,90.54,119.889	89.59,89.59,115.978	88.973,88.973,115.152	89.81, 89.81, 117.29
Total reflections	271,375 (19574)	137,590 (9996)	188672 (13965)	207720 (14821)
Unique reflections	13,589 (981)	13,688 (993)	18721 (1376)	10,342 (716)
Multiplicity	20.0 (2.0)	10.1 (10.1)	10.1 (10.2)	20.08 (14.44)
Completeness (%)	99.8 (98.4)	99.8 (99.1)	99.9 (99.8)	99.21 (93.2)
Mean I/sigma(I)	16.51 (0.7)	14.13 (0.77)	17.3 (0.7)	16.52 (0.42)
Wilson B-factor (Å^2)	104.75	104.24	95.50	146.86
CC1/2	1.0 (0.30)	1.0 (0.33)	1.0 (0.43)	1.0 (0.261)
CC*	1.0 (0.68)	1.0 (1.0)	1.0 (0.78)	1.0 (0.643)
R-work (%)	22.6 (35.2)	23.0 (50.0)	23.11 (36.9)	22.60 (42.65)
R-free (%)	28.0 (33.3)	27.5 (51.8)	28.32 (36.7)	29.90 (45.38)
Protein residues	400	403	403	404

RMS deviation bonds (Å)	0.005	0.006	0.007	0.012
RMS deviation angles (°)	0.72	0.91	0.95	1.31
Ramachandran plot favoured (%)	93.62	95.95	95.95	91.92
allowed (%)	6.12	3.54	3.54	6.57
outliers (%)	0.26	0.51	0.51	1.52
Rotamer outliers (%)	2.37	1.46	1.47	0.00
Clash score	15.74	12.75	14.07	15.16
Average B-factor ($Å^2$)	114.71	117.56	106.16	155.46

Supplementary Table 5. Mutations observed in *vapC*

Mutations observed in strains with low plasmid maintenance

Type of mutation	Position	Nucleotide change	Predicted amino acid change
duplication	300	GCCCGTCAGGGACGCCCTGTCGG GCCATTTGATCAAATG	Extra 12 amino acids
frameshift	319-320	AG	^{107}SGADYCD*
point	292	G -> T	D98Y
substitution	393	G -> A	W131*
substitution	364	C -> T	R108W
duplication/ frameshift	386	GAACTGA	^{130}N*
deletion/ frameshift	320-333	GTCGGGGGCTGATT	^{107}NCD*
deletion	232-243	ACACACACCGGC	^{76}AG^{81}
frameshift	232	A	^{76}AHTPAR*
substitution	3	ATG -> ATA	New start ^{4}M
substitution	151	CAG -> TAG	Q51*
substitution	361	GAA -> TAA	E120*
substitution	310	C -> A	H104N

Mutations observed in strains with high plasmid maintenance

substitution	322	C -> T	R122S

Supplementary Figure 1. Alignment of the nucleotide and amino acid sequences of VapBC from *S. sonnei* 53G and CS14.

Alignment of promoter and coding regions of *vapBC* in *S. sonnei* strains 53G and CS14. The two *vapBC* operator sites, OS1 and OS2 (orange), T to C substitution in the *vapBC* promoter region (grey), *vapB* coding region (blue), A to T substitution in the *vapB* open reading frame (dark pink), three phenylalanines (F, green), and the *vapC* coding region (pink).

Supplementary Figure 2. Effect of VapBC alleles and the promoter on plasmid loss from *E. coli.*

(**A**) Schematic of pSTAB2 containing the ori from pINV, *sac*:*kan* to select for plasmid absence/presence, and *vapBC*. (**B**) Plasmid loss assays conducted in *E. coli* MG1655 at 21 °C or 37 °C. Each dot represents the result for a single colony (n=9 for each strain). Each strain was tested on three independent occasions; horizontal line, mean. pSTAB2 expressing $VapB^{L12}C$ or $VapB^{A12}C$ under the control of either the *vapBC* promoter or the constitutive promoter, J23101 *vs.* $VapB^{Q12}C$ with the same promoter, p <0.0001, two-way ANOVA.

Supplementary Figure 3. **Structural comparison of VapBC DNA binding domains and EMSAs conducted with VapBC mutants.**

(**A**) Alignment of *S. sonnei* 53G VapBC (PDB 6SD6) with *Salmonella* VapBC bound to DNA (blue, space filled, PDB 6IFM). VapB and VapC are labelled, with only *S. sonnei* VapBC shown to enable visualisation of the VapB:DNA interaction. EMSAs demonstrating binding of $VapB^{Q12}C$ (**B**), $VapB^{L12}C$ (**C**), and $VapB^{A12}C$ (**D**) to DNA containing a 33 bp dsDNA single operator site, OS1. A 68 bp intergenic sequence was included as control DNA.

Supplementary Figure 4. Surface plasmon resonance of VapBC interacting with OS1.

Measurement of VapBC binding to OS1 by surface plasmon resonance for $VapB^{Q12}C$ (**A**), $VapB^{L12}C$ (**B**), and $VapB^{A12}C$ (**C**). (**D**) Estimation of K_D for VapBC binding to OS1.

Supplementary Figure 5. Quantification of degradation of VapBC by Lon.

(**A**) Quantification of *in vitro* proteolysis assays conducted at 37 °C with indicated VapBC proteins and purified *Shigella* Lon_6 protease with indicated combinations of Lon, ATP, and polyphosphate (polyP). (**B**) Assays were performed with the addition of *vapBC* promoter DNA or control DNA (indicated). Each proteolysis experiment was conducted on three separate occasions, and graphs show mean +/- SD.

Supplementary Figure 6. Mapping peptide sequences identified by MS after digestion of VapB.

$VapB^{Q12}C$ (**A**), $VapB^{L12}C$ (**B**) and $VapB^{A12}C$ (**C**) were subject to tryptic digestion with or without Lon digestion. Resulting peptides identified by MS were mapped onto the VapB amino acid sequences. Peptides present after tryptic digest are indicated in red, and non-tryptic peptides present after Lon digestion are indicated in blue. Lon digestion was carried out in the presence of polyphosphate.

Supplementary Figure 7. Manual analysis of MS spectra of a non-tryptic peptide present after Lon cleavage of VapBC.

Intact MS spectra of 2H+ and 3H+ charge states of non-tryptic peptide ion MDNREQPGMQERESF, present in VapBC samples incubated with Lon protease, but absent in VapBC control samples not digested by Lon protease.

Supplementary Figure 8. Manual analysis of MS spectra of a non-tryptic peptide present after Lon cleavage of VapBC.

Intact MS spectra of 2H+ and 3H+ charge states of non-tryptic peptide ion DGHSVSADFMDNREQPGMQERESF, present in VapBC samples incubated with Lon protease, but absent in VapBC control samples not digested by Lon protease.

Supplementary Figure 9. VapB tolerance mutations reduce interactions with the *vapBC* promoter.

(**A**) Spot assay of *E. coli* TB28 (MG1655 Δ*lacZYA*) transformed with pGH254 expressing *lacZYA* under the native *E. coli vapBC* promoter with pBAD33 expressing VapBC with different versions of VapB (indicated). Inhibition of the *lacZYA* locus (seen by white colonies) is only observed when wild-type *vapBC* (WT) is induced. Leaky expression of *vapC* from pBAD33 reduces bacterial survival under non-induced conditions. (**B**) Loss of pSTAB2 with different *vapBC* alleles from *S. flexneri* M90T or the isogenic Δ*clp* mutant. Plasmid loss was unaffected by absence of the Clp protease.

Supplementary Figure 10. Electron density around tolerance mutations in F plasmid VapB.

(**A**) Alignment of the structure of wild-type *E. coli* VapBC with $VapB^{T3N}C$, $VapB^{T3N+A13P+L16R}C$ and $VapB^{V5E}C$. All structures show a similar fold. (**B**) Electron Density in area around the T3N mutation of VapB. (**C**) Electron density around the area og the T3N+A13P+L16R mutations. (**D**) Electron density in the area around the V5E mutation of VapBC.

References

5 McVicker, G., Hollingshead, S., Pilla, G. & Tang, C. M. Maintenance of the virulence plasmid in Shigella flexneri is influenced by Lon and two functional partitioning systems. *Mol Microbiol* **111**, 1355-1366, doi:10.1111/mmi.14225 (2019).

19 McVicker, G. & Tang, C. M. Deletion of toxin-antitoxin systems in the evolution of Shigella sonnei as a host-adapted pathogen. *Nat Microbiol* **2**, 16204, doi:10.1038/nmicrobiol.2016.204 (2016).

36 Angelini, M., Stehling, E. G., Moretti, M. L. & da Silveira, W. D. Molecular epidemiology of Shigella spp strains isolated in two different metropolitam areas of southeast Brazil. *Braz J Microbiol* **40**, 685-692, doi:10.1590/s1517-838220090003000034 (2009).

56 Cherepanov, P. P. & Wackernagel, W. Gene disruption in Escherichia coli: TcR and KmR cassettes with the option of Flp-catalyzed excision of the antibiotic-resistance determinant. *Gene* **158**, 9-14, doi:10.1016/0378-1119(95)00193-a (1995).

75 Kopecko, D. J., Washington, O. & Formal, S. B. Genetic and physical evidence for plasmid control of Shigella sonnei form I cell surface antigen. *Infect Immun* **29**, 207-214, doi:10.1128/iai.29.1.207-214.1980 (1980).

76 Zychlinsky, A., Prevost, M. C. & Sansonetti, P. J. Shigella flexneri induces apoptosis in infected macrophages. *Nature* **358**, 167-169, doi:10.1038/358167a0 (1992).

77 Guyer, M. S., Reed, R. R., Steitz, J. A. & Low, K. B. Identification of a sex-factor-affinity site in E. coli as gamma delta. *Cold Spring Harb Symp Quant Biol* **45 Pt 1**, 135-140, doi:10.1101/sqb.1981.045.01.022 (1981).

78 Bærentsen, R. L. *et al.* Structural basis for kinase inhibition in the tripartite E. coli HipBST toxin–antitoxin system. *eLife* **12**, RP90400, doi:10.7554/eLife.90400 (2023).

79 Miroux, B. & Walker, J. E. Over-production of proteins in Escherichia coli: mutant hosts that allow synthesis of some membrane proteins and globular proteins at high levels. *J Mol Biol* **260**, 289-298, doi:10.1006/jmbi.1996.0399 (1996).

80 Hanahan, D. Studies on transformation of Escherichia coli with plasmids. *J Mol Biol* **166**, 557-580, doi:10.1016/s0022-2836(83)80284-8 (1983).

81 Wood, W. B. Host specificity of DNA produced by Escherichia coli: bacterial mutations affecting the restriction and modification of DNA. *J Mol Biol* **16**, 118-133, doi:10.1016/s0022-2836(66)80267-x (1966).

82 Datsenko, K. A. & Wanner, B. L. One-step inactivation of chromosomal genes in *Escherichia coli* K-12 using PCR products. *Proceedings of the National Academy of Sciences* **97**, 6640-6645, doi:doi:10.1073/pnas.120163297 (2000).
